# microRNA-193a stimulates pancreatic cancer cell repopulation and metastasis through modulating TGF-β2/TGF-βRIII signalings

**DOI:** 10.1186/s13046-018-0697-3

**Published:** 2018-02-13

**Authors:** Chi Fang, Chen-yun Dai, Zhu Mei, Ming-jie Jiang, Dian-na Gu, Qian Huang, Ling Tian

**Affiliations:** 1 Institute of Translational Medicine, Science bldg. Rm 205, Shanghai General Hospital, Shanghai Jiao Tong University School of Medicine, New Songjiang Rd No.650, Songjiang District, Shanghai, 201620 China; 2 Shanghai Key Laboratory of Pancreatic Diseases, Shanghai General Hospital, Shanghai Jiao Tong University School of Medicine, Shanghai, China; 3 Department of Gastroenterology, Shanghai General Hospital, Shanghai Jiao Tong University School of Medicine, Shanghai, China; 4 The Comprehensive Cancer Center, Shanghai General Hospital, Shanghai Jiao Tong University School of Medicine, Shanghai, China

**Keywords:** microRNA-193a, TGF-β2, TGF-βRIII, Cancer repopulation, Cancer metastasis, Pancreatic cancer

## Abstract

**Background:**

Pancreatic cancer characterizes high recurrence and poor prognosis. In clinical practice, radiotherapy is widely used for pancreatic cancer treatment. However, the outcome remains undesirable due to tumor repopulation and following recurrence and metastasis after radiation. So, it is highly needed to explore the underlying molecular mechanisms and accordingly develop therapeutic strategies. Our previous studies revealed that dying cells from chemoradiation could stimulate repopulation of surviving pancreatic cancer cells. However, we still knew little how dying cells provoke pancreatic cancer cell repopulation. We herein would explore the significance of TGF-β2 changes and investigate the modulation of microRNA-193a (miR-193a), and identify their contributions to pancreatic cancer repopulation and metastasis.

**Methods:**

In vitro and in vivo repopulation models were established to mimic the biological processes of pancreatic cancer after radiation. Western blot, real-time PCR and dual-luciferase reporter assays were accordingly used to detect miR-193a and TGF-β2/TGF-βRIII signalings at the level of molecular, cellular and experimental animal model, respectively. Flow cytometry analysis, wound healing and transwell assay, vascular endothelial cell penetration experiment, and bioluminescence imaging were employed to assessthe biological behaviors of pancreatic cancer after different treatments. Patient-derived tumor xenograft (PDX) mice models were established to evaluate the therapeutic potential of miR-193a antagonist on pancreatic cancer repopulation and metastasis after radiation.

**Results:**

miR-193a was highly expressed in the irradiated pancreatic cancer dying cells, accordingly elevated the level of miR-193a in surviving cells, and further promoted pancreatic cancer repopulation and metastasis in vitro and in vivo. miR-193a accelerated pancreatic cancer cell cycle and stimulated cell proliferation and repopulation through inhibiting TGF-β2/TGF-βRIII/SMADs/E2F6/c-Myc signaling, and even destroyed normal intercellular junctions and promoted metastasis via repressing TGF-β2/TGF-βRIII/ARHGEF15/ABL2 pathway. Knockdown of miR-193a or restoration of TGF-β2/TGF-βRIII signaling in pancreatic cancer cells was found to block pancreatic cancer repopulation and metastasis after radiation. In PDX models, the treatment in combination with miR-193a antagonist and radiation was found to dramatically inhibit pancreatic cancer cell repopulation and metastasis, and further improved the survival after radiation.

**Conclusions:**

Our findings demonstrated that miR-193a stimulated pancreatic cancer cell repopulation and metastasis through modulating TGF-β2/TGF-βRIII signalings, and miR-193a might be a potential therapeutic target for pancreatic cancer repopulation and metastasis.

**Electronic supplementary material:**

The online version of this article (10.1186/s13046-018-0697-3) contains supplementary material, which is available to authorized users.

## Background

Pancreatic cancer is one of the most known malignancies with a five-year survival rate of ~ 5% [1]. Despite of great efforts on treatment, the therapeutic outcomes remain quite poor to date. So far, radiotherapy has been widely used in clinical treatment of locally advanced or unresectable pancreatic cancer [2]. Although radiotherapy can relieve some clinical symptoms, it fails to exert survival benefits for patients due to tumor repopulation and following recurrence and metastasis [3, 4]. In fact, it has long been recognized that tumor repopulation often occurs after radiotherapy and chemotherapy, and causes treatment failure in varied tumors [5–7]. However, inhibition of repopulation could exert potentials for abrogation of cancer chemoradiation resistance [7]. Our earlier studies have revealed that dying cells from chemoradiation could directly stimulate repopulation of pancreatic cancer surviving cells by evoking the crosstalk between activated SHH and inactivated Wnt signaling [8], activating caspase-3/7-PKCδ signaling [9], and upregulating Sox2 [10]. Even so, we still knew little how dying cells provoke cancer repopulation after radiotherapy.

Particularly, TGF-β signaling was known to be one of 12 core signalings in pancreatic cancer [11] and involved in tumor radiation response [12]. Inhibition of endogenous TGF-β could enhance radiation resistance and improve cell proliferation, yet restoration of TGF-βcould enhance radiosensitivity in pancreatic cancer cells [13]. Hence, we wonder whether TGF-β signaling can regulate pancreatic cancer repopulation after radiotherapy.

Moreover, microRNAs (miRNAs) have been found to play important roles in tumor radiation response in recent years [14]. Certain miRNAs were confirmed to alter expression upon radiation [15]. Meanwhile, we have noted that some miRNAs are involved in the regulation of TGF-β signaling, such as miR-520c [16], miR-17-92 [17] and miR-106b-25 [18]. Accordingly, we conjectured that miRNAs might modulate pancreatic cancer repopulation after radiotherapy through regulating TGF-β signaling.

In fact, microRNA-193a (miR-193a) was found to be induced by radiation in glioma and cervical carcinoma [19], and contributed to chemoradiation resistance in oesophageal carcinoma [20]. Notably, we speculated that TGF-β2 might be the target of miR-193a according to the prediction of several software on miRNAs. Therefore, we conducted an Affymetrix Glue Grant Human Transcriptome Array to compare coding or non-coding RNA profiles between the irradiated and the untreated cancer cells. Coincidentally, TGF-β2 in the irradiated cancer cells was found to dramatically decrease compared with the untreated group. As known, TGF-β2 is one isoform of TGF-β family. Unlike TGF-β1 and TGF-β3 that can directly bind to TGF-β receptor II (TGF-βRII) and TGF-βRI, TGF-β2 shows very low affinity to TGF-βRII and TGF-βRI, and depends on TGF-βRIII assistance for transduction [21]. Virtually, abnormal TGF-β2 expression is more frequent in pancreatic cancer than the other TGF-β isoforms [22]. Moreover, TGF-β2 but not TGF-β1 or TGF-β3 was found to highly elevate in dormant cells of head and neck squamous cell carcinoma and induce cancer cell quiescence [23]. Remarkably, TGF-β2 could induce growth arrest through TGF-βRIII, and the disseminated tumor cells with high TGF-βRIII were prone to enter dormancy [23]. So, we wondered that TGF-β2/TGF-βRIII signaling might play a crucial role in pancreatic cancer upon chemoradiation.

Herein, we would explore the significance of TGF-β2 changes and investigate the elevation of miR-193a caused by ionizing radiation, and identify their contributions to pancreatic cancer repopulation. We found that the dying cells from chemoradiation would release highly elevated miR-193a, which further blockaded TGF-β2/TGF-βRIII signalings and stimulated the surviving tumor cell proliferation, and eventually caused pancreatic cancer repopulation and promoted tumor metastasis.

## Methods

### Cell culture

Human pancreatic cancer cell lines SW1990 (ATCC® CRL-2172™) and AsPC-1 (ATCC® CRL-1682™) were cultured in RPMI 1640 medium (HyClone) containing 10% fetal bovine serum (FBS, Gibco), 100 U/ml penicillin and 100 mg/ml streptomycin (HyClone) at 37 °C under 5% CO2. Human pancreatic cancer cell line PANC-1 (ATCC® CRL-1469™), human immortalized, nontumorigenic pancreatic ductal epithelial cell line HPDE6-C7 (Ontario cancer center, Canada), human umbilical vein endothelial cell line HUVEC (ATCC® PCS-100-010™) and human embryonic kidney cell line 293 T (ATCC® CRL-3216™) were cultured in DMEM (HyClone) containing 10% FBS (Gibco), 100 U/ml penicillin and 100 mg/ml streptomycin at 37 °C under 5% CO2.

### Oligonucleotides, constructs and lentiviruses

The 3’-UTR of TGF-β2 (1253 bp), TGF-βRIII (235 bp), ABL2 (407 bp), E2F6 (288 bp) and ARHGEF15 (360 bp) were all amplified from 293 T cells by RT-PCR (Life Technologies). The PCR primers (synthetized by Sangon Biotech, China) were provided in Additional file 1: Table S1. The amplified 3’-UTRs were confirmed by sequencing (Sangon Biotech, China), and further inserted into the reporter plasmid pmirGLO (7350 bp, Promega, USA) by double-digestion with SacI/XbaI (NEB). The resultant reporter plasmids were respectively designated as pmirGLO-TGF-β2–3’UTR (pmirGLO-TGB2U), pmirGLO-TGF-βRIII- (pmirGLO-TGBRIIIU), pmirGLO-TGF-ABL2–3’UTR (pmirGLO-ABL2U), pmirGLO-TGF-E2F6–3’UTR (pmirGLO-E2F6U), and pmirGLO-ARHGEF15–3’UTR (pmirGLO-AGEF15U). The putative counterparts (WTs and MTs) of miR-193a-target sequence in the 3’-UTR of TGF-β2 (GenBank NM_001135599), TGF-βRIII (GenBank, NM_003243), ABL2 (GenBank, NM_005759), E2F6 (GenBank, NM_001278278) and ARHGEF15 (GenBank, NM_025014) were synthetized by Sangon Biotech (Shanghai, China) and also inserted into the same reporter vector, and named as pmirGLO-TGB2U-WT, pmirGLO-TGB2U-MT, pmirGLO-TGBRIIIU-WT, pmirGLO-TGBRIIIU-MT, pmirGLO-ABL2U-WT, pmirGLO-ABL2U-MT, pmirGLO-E2F6U-WT, pmirGLO-E2F6U-MT, pmirGLO-AGEF15U-WT and pmirGLO-AGEF15U-MT, respectively. The synthesized sequences were shown in Additional file 1: Table S2. The schematic physical maps of these constructs were shown in Additional file 2: Figure S1.

The pri-miR-193a was also amplified from 293 T genomic DNA by PCR (Life Technologies), and the PCR primers (synthetized by Sangon Biotech, China) were provided in Additional file 1: Table S1. The amplified pri-miR-193a was confirmed by sequencing (Sangon Biotech, China), and further cloned into the expression plasmid of pLEX-EgmiR-CvG2L vector [24] by double-digestion with BamHI/NheI (NEB). The corresponding recombinant vector is designated as pLEX-EgmiR-193a/CvG2L. The sequence of miR-193a inhibitor (miR-193a-IN) was synthetized by Sangon Biotech (Shanghai, China) and was cloned into the vector pLV-hU6shRNA/CvG2L (10,704 bp). The corresponding recombinant vector are designated as pLV-hU6miR-193a-IN/CvG2L. These constructs were schematically shown in Additional file 3: Figure S2.

Recombinant lentiviruses were produced by co-transfecting 293 T cells with the expression vectors pLEX-EgmiR-CvG2L, pLEX-EgmiR-193a/CvG2L, pLV-hU6shRNA/CvG2L or pLV-hU6miR-193a-IN/CvG2L and the packaging and envelope plasmids pMD2.G (Addgene #12259), psPAX2 (Addgene #12260). The corresponding recombinant viruses were designated as LV-EgmiR-NC, LV-EgmiR-193a, LV-hU6shR-NC and LV- hU6miR-193a-IN, respectively. Viruses were harvested at 48 h and 72 h by filtering the virus-containing medium through 0.45 μm Steriflip fliter (Millipore, USA) for future use.

### Development of stable pancreatic cancer cells

The firely luciferase (Fluc) and green fluorescent protein (GFP) fusion gene, mcherry fluorescent protein (mcherry) gene are preserved in our lab. They were inserted into the lentiviral vector pLEX-MCS (Thermo Scientific, USA). All of live, replication-deficient recombinant lentiviral vectors were packaged in 293 T cells using Lipofectamine 2000 (Invitrogen, USA) following manufacturer’s instructions. Viruses were harvested at 48 h and 72 h by filtering the virus-containing medium through 0.45 μm Steriflip fliter (Millipore, USA). Virus infection was performed in the indicated cells with 8 μg/ml polybrene (Sigma-Aldrich, USA). The transfected stable cells were sorted by MoFlo High-Performance Cell sorter (Beckman Coulter, USA), and designated as SW1990-EgmiR-193a, SW1990-EgmiR-NC, PANC-1-hU6miR-193a-IN, and PANC-1-hU6shR-NC, respectively. Accordingly, the luciferase/GFP-labeled cells were named as SW1990-G2L, PANC-1-G2L and HUVEC-G2L. The mcherry-labeled cells were named as SW1990- mcherry and PANC-1- mcherry.

### RNA extraction and real-time PCR

Total RNAs were extracted using TRIzol Reagent (TakaRa, Japan), and reverse transcribed into complementary DNA using PrimeScript™ RT reagent kit (TakaRa, Japan). Real-time PCR was performed using SYBR Premix Ex Taq II (Takara, Japan) on the QuantStudio 6 Flex (Life technologies, USA). The primers of miR-193a and the control of U6 snRNA (MQP-0201) were purchased from RiboBio (Guangzhou, China). The 2^-△△CT^ method was used to determine fold changes. The basal miR-193a expression in multiple pancreatic cells was tested (Additional file 4: Figure S3A).

### Western blot

Cells were routinely harvested as indicated time, and the irradiated cells were harvested at 24 h after radiation. Protein lysates were separated in SDS-PAGE, and transferred to PVDF membranes (Millipore, USA). The membranes were blocked with 5% non-fat milk in TBS buffer for 1 h, and then incubated with primary antibodies overnight at 4 °C. At room temperature, the membranes were incubated with HRP-labeled secondary antibodies (Sigma, USA) for 2 h. The reaction was visualized using Immobilon™ Western HRP substrate kit (Millipore, USA). The primary antibodies were as follows: TGF-β2 and c-Myc (Santa Cruz, USA), TGF-βRIII (CST, USA), ARHGEF15 (Abcam, USA), ABL2 (Abcam, USA), p-SMAD2/3 (Abcam, USA), E2F6 (Abcam, USA), SMAD4 (Abcam, USA), GAPDH (CST, USA). SMAD4 protein expression in pancreatic cells was tested by western blot (Additional file 4: Figure S3B).

### Dual-luciferase reporter assay

293T cells were cultured in 96-well plates, and co-transfected with the reporter plasmids and miR-193a angomirs (RiboBio, China) using Lipofectamine 2000 (Invitrogen, USA). Luciferase assays were conducted at 48 h of post-transfection using Dual-Luciferase®Reporter Assay (Promega, USA) according to the manufacturer’s protocol. The *Renilla* and firefly luciferase activities were measured, and the ratio was calculated. The experiments were repeated for three times.

### Immunofluorescence staining

The cultured cells were routinely harvested as indicated time. Cells were fixed with 4% paraformaldehyde and then permeabilized with 0.1% Triton X-100. After treatment with blocking buffer, cells were incubated with primary antibody E-cadherin and N-cadherin (CST, USA) at 4 °C overnight. At room temperature, cells were incubated with fluorescein-labeled secondary antibody (CST, USA) for 2 h. Cells were counterstained with DAPI. Immunofluorescence was visualized by confocal microscope (Leica TCS SP8, Germany).

### Flow cytometry

Cells were routinely cultured as indicated conditions. The cells were trypsinized, and further collected to be fixed in 75% ethanol at − 2 °C for 24 h. Cells were stained using BD Pharmingen™ PI/ RNase staining (BD, USA). Cell cycle was measured using Accuri C6 Flow Cytometer (BD, USA). The data were analyzed using BD Accuri C6 software and ModFit LT software.

### Wound healing assay

The stable cells, SW1990-EgmiR-193a, SW1990-EgmiR-NC, PANC-1-hU6shR-NC and PANC-1-hU6miR-193a-IN, were seeded in 6-well plates. The linear wound was made when the cell confluence reached 80–90% using 10 μl tips. The linear wound was observed and photographed at 0 h, 36 h and 48 h under the microscopy (Leica, Germany). The statistic quantification has been made using Image J software.

### Transwell assay

Cells were cultured in the hanging cell culture inserts of 8 μm pore size (PIEP12R48, Millipore) for 24-well plates. 200 μl fresh medium containing 2% FBS was added to the hanging cell culture inserts. 900 μl fresh medium containing 10% FBS was added to the lower chamber. After 24 h, the transmigrated cells were fixed with 4% paraformaldehyde, and stained with crystal violet. Cells in the inserts were removed with cotton swabs. Representative images were observed and photographed under the microscopy (Leica, Germany).

### Vascular endothelial cell penetration experiment

The vascular endothelial cell penetration experiment was performed according to the manufacture’s protocol (Glycotech, USA). In brief, the basal cells HUVEC-G2L were cultured on the slides coated with matrigel matrix (BD, USA). The co-cultured reporter cells of SW1990-mcherry and PANC-1-mcherry with corresponding feeder cells (SW1990 and PANC-1, non-treatment or X-ray) were used for the flow cells. The parallel plate flow chamber (Glycotech, USA) was used for flow assay. The flow speed was about 5 ml every hour, and kept for 2 h. 1 day after flow assay, the penetration state was observed by confocal microscope (Leica, TCS SP8, Germany).

### Bioluminescence imaging

Luciferase signals were from D-luciferin (Promega, USA) using the indicated concentration according to the manufacturer’s instructions. Bioluminescence imaging of cells and mice was performed in the IVIS Lumina Series III (PerkinElmer, USA). The luciferase signal activity was measured and analyzed quantitatively using the manufacturer supplied software. The bioluminescent images of repopulation model in vitro were taken through a confocal microscope from Leica Microsystems (TCS SP8, Germany).

### In vitro repopulation model

Pancreatic cancer cells were irradiated with 10Gy using an Oncor linear accelerator (Siemens, Germany) in our hospital. The dose rate is about 3.6Gy/min. Pancreatic cancer cells (feeder cells) were seeded into the culture plate overnight with 2% FBS in culture medium before radiation. Luciferase/GFP-labeled or mcherry-labeled living pancreatic cancer cells (reporter cells) were immediately seeded into the co-culture system after radiation. The ratio of feeder cells and reporter cells was 100:1. The fresh culture medium containing 2% FBS was regularly replaced every 2 days for 2 weeks. Tumor cell repopulation was measured by bioluminescence imaging. Representative fluorescent images were taken under confocal microscope (Leica, Germany).

### Animal experiments of tumor models

BALB/c nude mice (6 weeks) were purchased from Shanghai Sippr-BK laboratory animal Co. Ltd. (certificate #SCXK (Shanghai) 2013–0016). Pancreatic cancer cells were resuspended in 100 μl free serum medium and injected subcutaneously into the right forelimb of nude mice. The length and the width of tumor were measured every two or three days for 5–7 weeks. Tumor volume was calculated using the formula (Volume = 0.5 × length × width^2^). The mice were sacrificed 34 days after injection for in vivo repopulation model, around 30 days for in vivo proliferation and metastasis assay, and about 75 days for in vivo survival assay, respectively. Mice were anesthetized with isoflurane gas, and sacrificed by cervical dislocation. Tumor tissues and livers of these mice were collected, total RNA and protein were extracted from the tumor tissues for further tests.

### Patient-derived xenograft model

The cryopreserved pancreatic adenocarcinoma tissues (3rd passage) in our lab [24] were subcutaneously engrafted into the right anterior axillary fossa of BALB/c nude mice (6 weeks). When it grew to ~ 1.5cm^3^ volume, tumor was removed and once again subcutaneously engrafted into nude mice (6 weeks). The length and the width were measured every two or three days for ~ 6 weeks. Tumor volume was calculated using the formula (volume = 0.5 × length × width^2^). When tumor volume reached ~500mm^3^, miR-193a antagonist (RiboBio, China) was used for intra-tumor injection of the irradiated mice. Every mouse was injected 10 nmol miR-193a antagonist in 100 μl volume at twice one week for 4 weeks. The mice were sacrificed around 38 days after injection for in vivo proliferation, repopulation and metastasis assay, and around 50 days for in vivo survival assay, respectively. Tumor tissues and livers were obtained from the mice for further tests.

### X-ray radiation of tumor-bearing mice

Mice were administered radiation when tumor grew to approximately 200mm^3^ for the tumor-bearing mice loaded with SW1990-G2L and PANC-1-hU6shR-NC cells, whereas ~100mm^3^ for that with PANC-1-hU6miR-193a-IN cells. Two groups of tumor-bearing mice with PANC-1-hU6shR-NC and PANC-1-hU6miR-193a-IN cells were treated X-ray radiation, yet one group tumor-bearing mice with PANC-1-hU6shR-NC were untreated. For PDX mice, mice were administered radiation when the tumor volume was around 500mm^3^. Two groups of PDX mice were treated X-ray radiation. The X-ray dose was 10Gy using an Oncor linear accelerator (Siemens, Germany) in our hospital. The dose rate of the machine is about 3.6Gy/min.

### Statistical analysis

All data were analyzed with software GraphPad Prism 6. Normally distributed data were presented as mean ± SD. Differences between means were assessed using unpaired *t* test. *p* < 0.05 was considered statistically significant.

## Results

### The elevated miR-193a from irradiated dying cells promotes accelerated repopulation and metastasis of surviving cancer cells

**Fig. 1:**
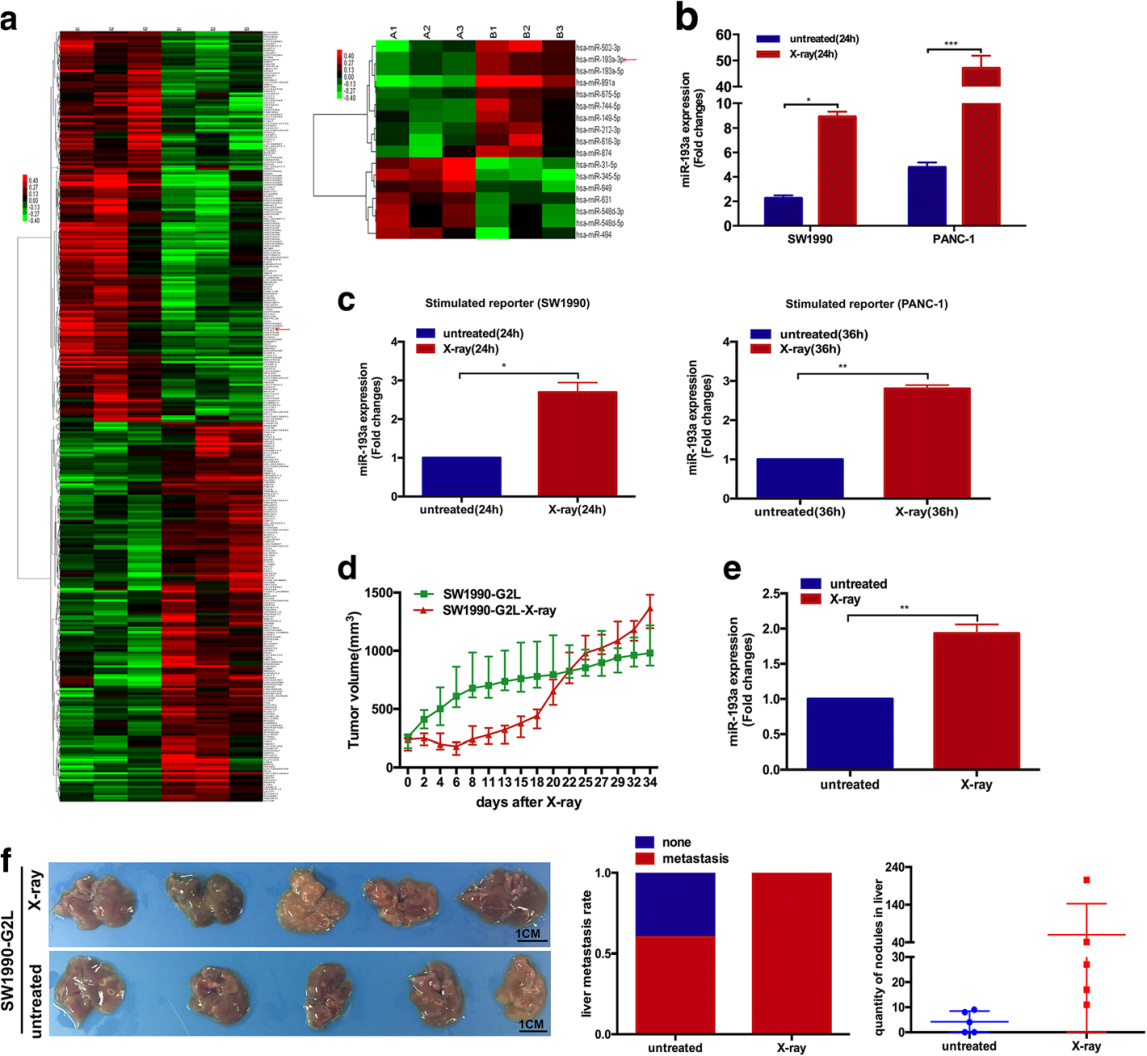
miR-193a from the irradiated cells promotes cancer repopulation and metastasis. **a** Heatmap of mRNA (*Left*) and miRNA (*Right*) profiles in the untreated (A1-A3) and the irradiated (B1-B3) cancer cells. **b** & **c** RT-qPCR for detecting miR-193a expression changes in pancreatic cells (±X-ray), and in the stimulated SW1990 and PANC-1 reporter cells co-cultured with their corresponding feeder cells (±X-ray). ***p < 0.001, **p < 0.01, *p < 0.05. *n* = 3. **d** Tumor growth curve of pancreatic cancer (burdened with 5 × 10^6^ SW1990-G2L cells) on X-ray radiation. *n* = 5. (E) RT-qPCR for assessing miR-193a expression changes in mice (±X-ray) with pancreatic tumors. **p < 0.01. (F) Liver metastasis of tumor-bearing mice (±X-ray) with SW1990-G2L cells. *Left*, photograph of the dissected liver, *n* = 5. Scale bar, 1 cm. *Central*, liver metastasis rate. *Right*, numbers of liver nodules

Pancreatic cancer repopulation was observed in clinical radiotherapy, and we have ever explored it in vitro and in vivo [7–10]. Hereinafter, we would investigate whether miRNAs were involved. We first employed the Affymetrix Glue Grant Human Transcriptome Array (GG-H array) 1.0 to analyze the expression profiles of coding or non-coding RNAs between the irradiated and the untreated cancer cells (GEO accession number GSE104965). Clear distinction of the expression profiles was shown in the heatmaps. Remarkably, TGF-β2 in the irradiated cancer cells was found to dramatically decrease compared with the untreated group (Fig. 1a, *Left*). Meanwhile, miR-193a expression was also upregulated (Fig. 1a, *Right*).

We hence established in vitro repopulation models, the detached co-culture system and the osculatory one, to examine these changes (Additional file 5: Figure S4). We found that miR-193a expression was upregulated in the irradiated SW1990 and PANC-1 cells compared with the untreated ones (Fig. 1b). Furthermore, higher miR-193a expression was observed in the stimulated SW1990 and PANC-1 reporter cells co-cultured with corresponding irradiated feeder cells than in those with untreated ones (Fig. 1c). These data indicated that the elevated miR-193a from the irradiated dying pancreatic cancer cells caused miR-193a increased in the living pancreatic cancer cells and promoted tumor repopulation in vitro*.*

We further explored whether miR-193a expression changed in vivo. Pancreatic cancer cell SW1990-G2L was subcutaneously injected into nude mice (Additional file 5: Figure S4D & S4E). We found that the tumor volume of these model mice presented dynamic changes (Fig. 1d). 3 days after irradiation, the tumor (~220mm^3^) became to shrink until it reduced to the minimum (~175mm^3^) at day 6. Then the irradiated tumor recovered and grew up slowly. About 3 weeks later, the irradiated tumor growth suddenly accelerated, the tumor volume even exceeded the untreated one, and the growth pace was much faster than before. Contrarily, the untreated tumor kept steady growth-up. Accordingly, miR-193a expression was detected in the tissues of the irradiated and the untreated tumor, and found to increase in the irradiated tumors (> 1.5-fold changes, Fig. 1e). Notably, a higher rate of spontaneous liver metastasis was found in the irradiated mice (~ 100%) than the untreated group (60%) (Fig. 1f, *Left* & *central*). Moreover, the number of metastatic nodules in the irradiated group (> 50 at average) were dramatically more than the untreated one (< 10 at average) (Fig. 1f, *Right*). These results showed that the irradiated dying pancreatic cancer promoted accelerated repopulation and metastasis of the surviving pancreatic cancer cells. Together, miR-193a was highly expressed in irradiated pancreatic cancer tissues, and might stimulate pancreatic cancer repopulation and promote metastasis.

### miR-193a accelerates cancer cell cycle, and promotes cell proliferation and repopulation

**Fig. 2:**
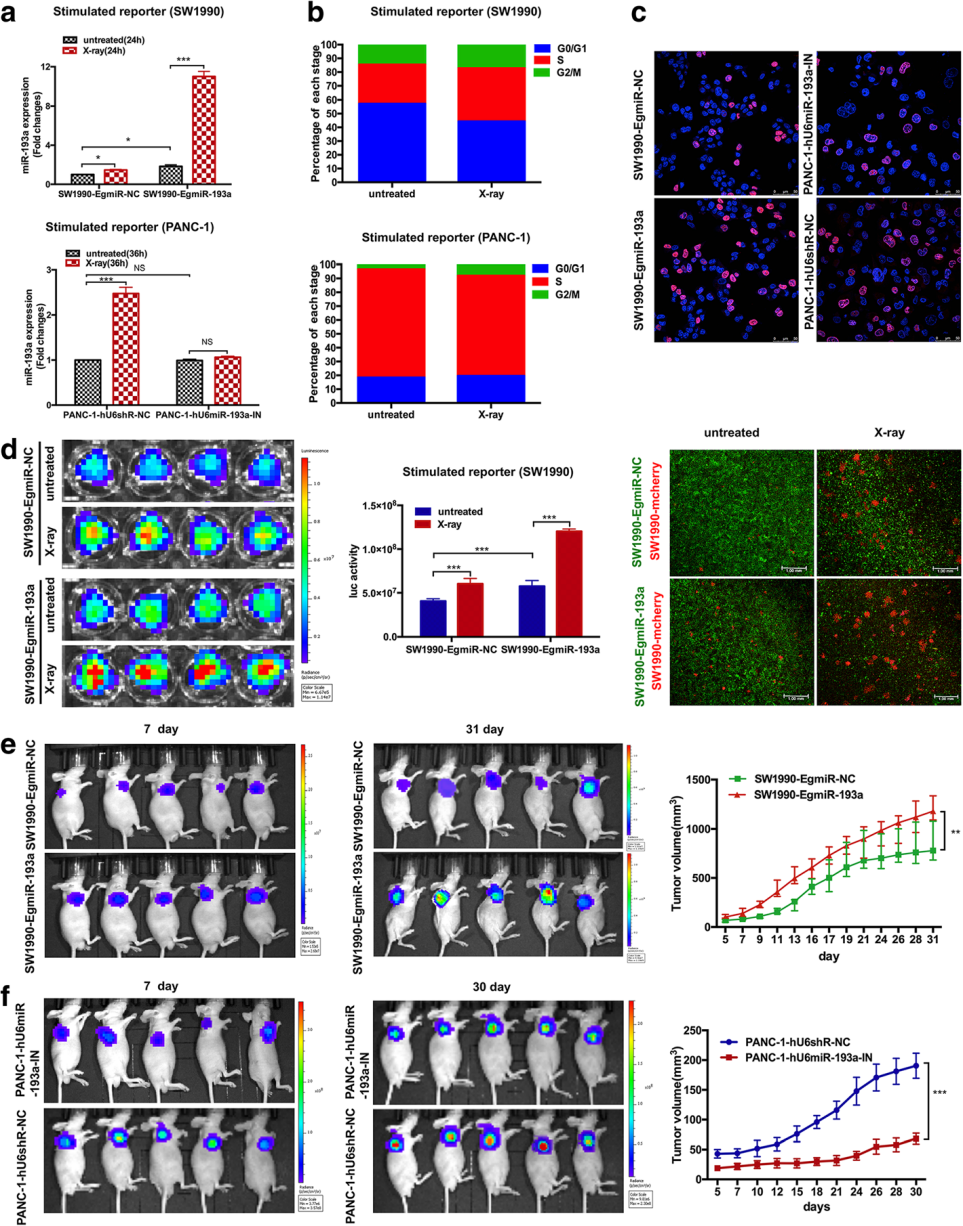
miR-193a accelerates cancer cell cycle and promotes cell proliferation and repopulation. **a** RT-qPCR for detecting miR-193a expression changes in the stimulated SW1990 and PANC-1 reporter cells co-cultured with their corresponding feeder cells (±X-ray). *p < 0.05, ***p < 0.001. n = 3. **b** Flow cytometry for assessing cell cycle in indicated cells. **c** EdU assay for detecting cell proliferation. Red, cells in proliferation stage. Blue, DAPI. Scale bar, 50 μm. **d** Repopulation of SW1990 reporter cells co-cultured with the corresponding feeder cells (±X-ray). *n* = 4. *Left*, representative bioluminescence images. *Central*, luciferase activity (photons/s). *Right*, representative fluorescent images. Scale bar, 1 mm. ***p < 0.001. **e** & **f**) miR-193a influenced pancreatic cancer cell proliferation in vivo. Nude mice were subcutaneously injected with SW1990-EgmiR-193a (E, 5 × 10^6^) and PANC-1-hU6miR-193a-IN cells (F, 8 × 10^6^). n = 5. *Left* & *central*, representative bioluminescence images in indicated time. *Right*, tumor growth curve of pancreatic cancer. ***p < 0.001, **p < 0.01

We further developed stable SW1990 cells with overexpression of miR-193a (SW1990-EgmiR-193a) (> 200-fold changes, Additional file 6: Figure S5A, *Left*), and PANC-1 cells with knockdown of miR-193a (PANC-1-hU6miR-193a-IN) (< 0.1-fold changes, Additional file 6: Figure S5A, *Right*) for analysis of their biological effects. Firstly, these stable cells served as feeder cells, and were treated with or without X-ray, and then co-cultured with the corresponding reporter cells. We found that higher miR-193a occurred in the stimulated SW1990-reporter cells co-cultured with the irradiated feeder cells than that of the controls (Fig. 2a, *Upper*). Obvious increase of miR-193a expression was also observed in the co-culture system with the irradiated SW1990-EgmiR-193a feeder cells (> 10-fold changes). Moreover, higher miR-193a expression was detected in the stimulated PANC-1-reporter cells co-cultured with the irradiated PANC-1-hU6shR-NC feeder cells than those with untreated PANC-1-hU6shR-NC ones (Fig. 2a, *Lower*). However, no significant alterations of miR-193a expression was observed in the stimulated PANC-1-reporter cells co-cultured with PANC-1-hU6miR-193a-IN feeder cells before and after radiation.

To further explore the effects of miR-193a in pancreatic cancer repopulation, we isolated and analyzed the reporter cells in vitro. We found that cell cycle of the stimulated SW1990- and PANC-1-reporter cells speeded up compared with those co-cultured with the corresponding untreated feeder cells (SW1990 and PANC-1, treated with or without X-ray) (Fig. 2b & Additional file 6: Figure S5B). The EdU assay showed that overexpression of miR-193 promoted pancreatic cancer cell proliferation, and vice versa (Fig. 2c). We then monitored the growth of stimulated reporter cells, and found that the luciferase activity increased in the stimulated SW1990-reporter cells co-cultured with the irradiated SW1990-EgmiR-NC ones, and in the SW1990-EgmiR-193a feeder cells (untreated or irradiated) compared with those untreated SW1990-EgmiR-NC ones. The highest luciferase activity was observed in the stimulated SW1990-reporter cells co-cultured with the irradiated SW1990-EgmiR-193a feeder cells (Fig. 2d, *Left* & *central*). In addition, in the osculatory co-culture model, feeder cells apparently diminished in the irradiated wells, and SW1990-mcherry reporter cells propagated in the wells seeded with the irradiated SW1990-EgmiR-NC, untreated or irradiated SW1990-EgmiR-193a feeder cells (Fig. 2d, *Right*).

We further explored the effects of miR-193a on pancreatic cancer cell proliferation in vivo. The luciferase/GFP-labeled pancreatic cancer cells (with or without miR-193a overexpression or knockdown) were subcutaneously injected into nude mice. We found that the SW1990-EgmiR-193a cell-loaded tumor grew faster, and the tumor volume was much larger than that of SW1990-EgmiR-NC cells (Fig. 2e). Moreover, knockdown of miR-193a in PANC-1 cells obviously slowed down cell growth, and resulted in a significant decrease in tumor volume (Fig. 2f). These results indicated that miR-193a could accelerate pancreatic cancer cell cycle, and promote cell proliferation and repopulation in vitro and in vivo*.*

### miR-193a promotes cancer repopulation through modulating TGF-β2/TGF-βRIII/SMADs/E2F6/c-Myc signaling

**Fig. 3:**
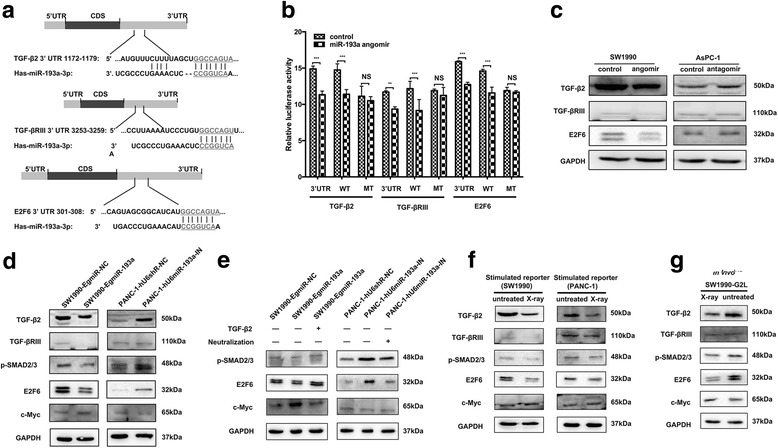
miR-193a promotes cancer repopulation by regulating TGF-β2/TGF-βRIII/SMADs/E2F6/c-Myc signaling. **a** Schematic diagrams of the predicted miR-193a-binding sites in the 3’-UTR of TGF-β2, TGF-βRIII and E2F6. **b** The relative luciferase activity on 3’-UTRs, WT and MT counterparts were measured. **p < 0.01, ***p < 0.001. n = 3. **c** Western blot for detecting TGF-β2, TGF-βRIII and E2F6 proteins in SW1990 cells transfected with miR-193a angomir, and AsPC-1 cells transfected with miR-193a antagomir. **d**-**g** Western blot for detecting TGF-β2/TGF-βRIII/SMADs/E2F6/c-Myc signaling respectively in SW1990-EgmiR-193a and PANC-1-hU6miR-193a-IN cells (**d**), in SW1990-EgmiR-193a (±TGF-β2 cytokine, 10 ng/ml) and PANC-1-hU6miR-193a-IN cells (±TGF-β2 neutralization antibody, 2.5 μg/ml) (**e**), in stimulated SW1990 and PANC-1 reporter cells co-cultured with the corresponding feeder cells (±X-ray) (**f**), and in tumor tissues from burdened-mice (±X-ray) with SW1990-G2L cells (**g**)

We further explored the underlying mechanisms of miR-193a on pancreatic cancer cell proliferation and repopulation. Intriguingly, miR-193a-binding sites were found in the 3’-UTR of TGF-β2 and its indispensable receptor TGF-βRIII according to the prediction of TargetScanHuman software (http://www.targetscan.org/) (Fig. 3a, *Upper* & *middle*). This coincidence might portend that miR-193a would mediate repopulation after radiation through modulating of TGF-β2/TGF-βRIII signaling. Notably, E2F6 was also similarly predicted as the target of miR-193a (Fig. 3a, *Lower*). As known, E2F6 could behave as a dominant-negative repressor to inhibit E2F- and Myc-responsive genes [25, 26].

Accordingly, the 3’-UTR of TGF-β2, TGF-βRIII and E2F6, as well as their corresponding miR-193a-binding sequences (WT) and mutant counterparts (MT) were cloned into the reporter plasmids, and the relative luciferase activities were further assessed. The dual-luciferase reporter assay showed that the relative luciferase activities were decreased in the 3’-UTR of TGF-β2, TGF-βRIII and E2F6 compared with the control group when miR-193a angomirs were exposed in 293 T cells. Similar results were observed to the WTs of TGF-β2, TGF-βRIII and E2F6. Nevertheless, there were no significant changes in the MTs of TGF-β2, TGF-βRIII and E2F6 (Fig. 3b). Further, ectopic expression of miR-193a in SW1990 cells transfected with miR-193a angomir reduced the protein expression of TGF-β2, TGF-βRIII and E2F6, while inhibition of miR-193a in AsPC-1 cells transfected with miR-193a antagomir could increase the expression of these proteins compared to the controls (Fig. 3c). These results suggested that TGF-β2, TGF-βRIII and E2F6 were the targeted genes of miR-193a.

So we further investigated the relationship between miR-193a and TGF-β2 signalings. As known, the phosphorylation of SMAD2/3 usually presented the activation of TGF-β2 signaling [27]. Besides, c-Myc was regulated by E2F6 and closely associated with cell cycle and tumorigenesis [28]. It was found that relatively lower expression of TGF-β2, TGF-βRIII, p-SMAD2, p-SMAD3, and E2F6, and higher expression of c-Myc was measured in SW1990-EgmiR-193a cells than in SW1990-EgmiR-NC ones, yet it was contrary in PANC-1-hU6miR-193a-IN compared to PANC-1-hU6siR-NC cells (Fig. 3d). Furthermore, restoration of TGF-β2 in SW1990-EgmiR-193a cells could abolish the inhibition of p-SMAD2, p-SMAD3, E2F6 and the increase of c-Myc expression caused by ectopic expression of miR-193a. Rather, neutralizing TGF-β2 in PANC-1-hU6miR-193a-IN cells could abrogate the increased expression of p-SMAD2, p-SMAD3, E2F6 and the decreased expression of c-Myc caused by knockdown of miR-193a (Fig. 3e).

We further explored whether miR-193a promoted pancreatic cancer repopulation through TGF-β2/TGF-βRIII signalings. Relatively lower expression of TGF-β2, TGF-βRIII, p-SMAD2, p-SMAD3 and E2F6, and higher level of c-Myc were detected in both SW1990- and PANC-1-reporter cells co-cultured with irradiated feeder cells than those with untreated ones (Fig. 3f). Likewise, tumor tissues were collected from model mice and used to test the expression of TGF-β2 signalings. Relatively lower level of TGF-β2, TGF-βRIII, p-SMAD2, p-SMAD3 and E2F6, and higher expression of c-Myc were detected in the irradiated tumors than in the untreated ones (Fig. 3g). Together, we believed that miR-193 promoted cell proliferation and accelerated repopulation through TGF-β2/TGF-βRIII/SMADs/E2F6/c-Myc signaling.

### miR-193a destroys intercellular junctions and promotes metastasis through repressing TGF-β2/TGF-βRIII/ARHGEF15/ABL2 signaling

**Fig. 4:**
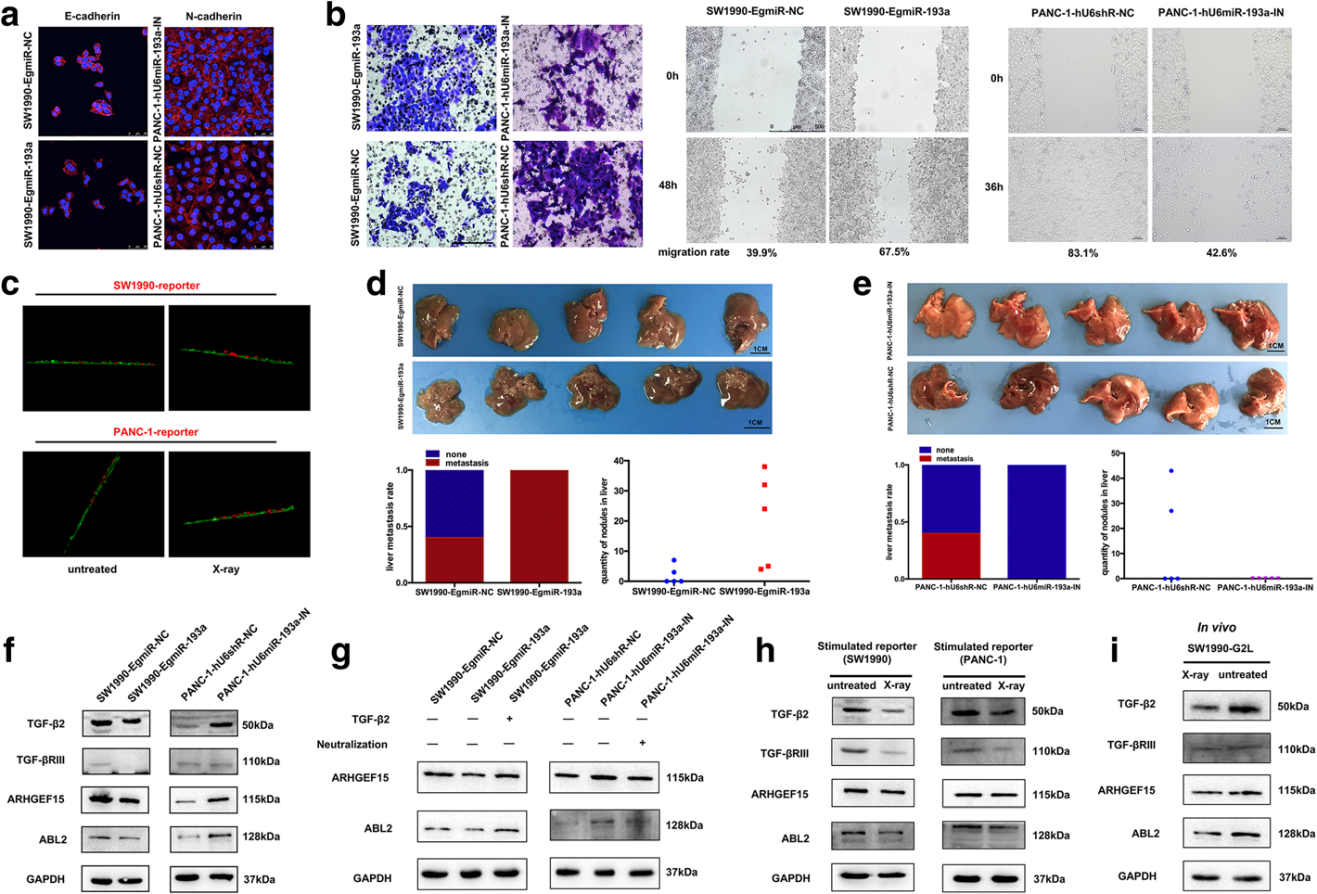
miR-193a destroys intercellular junctions and promotes cancer metastasis. **a** Immunofluorescence assay for observing E-cadherin and N-cadherin expression. Scale bar, 50 μm. **b** Transwell (*Left*, scale bar, 200 μm) and wound healing (*Central* & *right*, scale bar, 100 μm) assays for observing migration. **c** The penetration state of the stimulated SW1990 and PANC-1 reporter cells co-cultured with their corresponding feeder cells (±X-ray). Green cell (basal layer cell), HUVEC-G2L; Red cell (flow cell); SW1990-mcherry and PANC-1-mcherry reporter cells. **d** & **e** Liver metastasis of tumor-bearing mice with SW1990-EgmiR-193a cells **d** and PANC-1-hU6miR-193a-IN cells **e**. *Upper*, photograph of the dissected liver, n = 5. Scale bar, 1 cm. *Lower left*, liver metastasis rate. *Lower right*, numbers of liver nodules. *p < 0.05. **f**-**i** Western blot for testing TGF-β2/TGF-βRIII/ARHGEF15/ABL2 signaling in indicated cells


As described above, we found that miR-193a promoted pancreatic cancer repopulation after radiation. We wondered whether miR-193a could affect the intercellular junctions and further promote metastasis. It was found that ectopic expression of miR-193a could significantly decrease E-cadherin and N-cadherin expression, and vice versa (Fig. 4a). The wound healing and transwell assays showed that SW1990-EgmiR-193a cells displayed stronger potentials in migration and metastasis than that of SW1990-EgmiR-NC ones, yet contrary to that of PANC-1-hU6miR-193a-IN compared to PANC-1-hU6shR-NC cells (Fig. 4b). Furthermore, the vascular endothelial cell penetration experiment revealed that SW1990-mcherry or PANC-1-mcherry reporter cells co-cultured with the corresponding irradiated feeder cells exhibited greater capability in the traverse of HUVEC-G2L cells than that of the controls (Fig. 4c & Additional file 7: Video S1, Additional file 8: Video S2, Additional file 9: Video S3, Additional file 10: Video S4). In vivo, we observed metastasis in tumor-bearing mice with SW1990-EgmiR-193a (Fig. 4d) and PANC-1-hU6miR-193a-IN cells (Fig. 4e). As shown, most SW1990-EgmiR-193a cell-burdened mice, with cachexia performance, were more emaciated than those with SW1990-EgmiR-NC cells. The exploratory laparotomy revealed that a higher rate of spontaneous liver metastasis was in SW1990-EgmiR-193a cell-burdened mice than that of the controls (Fig. 4d, *Upper* & *lower left*). Even more, liver metastasis in the SW1990-EgmiR-193a-cell-burdened mice reached 100%. Moreover, the metastatic nodular number in the SW1990-EgmiR-193a-cell-burdened mice (> 20 at average) was more than that of the controls (< 10 at average) (Fig. 4d, *Lower right*). Conversely, lower rate of spontaneous liver metastasis in PANC-1-hU6miR-193a-IN-cell-burdened tumor mice was presented than that of the controls (Fig. 4e).

**Additional file 7: Video S1. **The penetration state of stimulated SW1990 reporter cells co-cultured with untreated SW1990 feeder. (MP4 2021 kb)



**Additional file 8: Video S2. **The penetration state of stimulated SW1990 reporter cells co-cultured with irradiated SW1990 feeder cells. (MP4 1121 kb)



**Additional file 9: Video S3. **The penetration state of stimulated PANC-1 reporter cells co-cultured with untreated PANC-1 feeder cells. (MP4 821 kb)



**Additional file 10: Video S4. **The penetration state of stimulated PANC-1 reporter cells co-cultured with irradiated PANC-1 feeder cells. (MP4 1199 kb)


We accordingly investigated the underlying mechanisms of miR-193a in pancreatic cancer metastasis. We thought miR-193a might promote metastasis through repressing TGF-β2/TGF-βRIII/ARHGEF15/ABL2 signaling. As known, Rho guanine nucleotide exchange factor 15 (ARHGEF15) functions as a specific guanine nucleotide exchange factor for Rho GTPase family [29], and involves in cell migration [30]. Thereinto, RhoA, a typical member of Rho GTPase family, plays a fundamental role in cell-cell junctional adhesion [31]. Moreover, RhoA could be regulated by TGF-β and involves in EMT [32]. Besides, ABL2, a cytoplasmic tyrosine kinase, can promote the intercellular adherens formation, yet inhibition of ABL2 causes normal intercellular junction breakdown [33]. Similarly, ARHGEF15 and ABL2 were also confirmed to be the targets of miR-193a (Additional file 11: Figure S6).

Moreover, relatively lower expression of TGF-β2, TGF-βRIII, ARHGEF15 and ABL2 was shown in SW1990-EgmiR-193a cells than in SW1990-EgmiR-NC ones, and vice versa in PANC-1-hU6miR-193a-IN cells compared to PANC-1-U6siR-NC ones (Fig. 4f). Further, restoration of TGF-β2 in SW1990-EgmiR-193a cells could abolish the repressed expression of ARHGEF15 and ABL2 caused by ectopic overexpression of miR-193a. Rather, neutralizing TGF-β2 in PANC-1-hU6miR-193a-IN cells could abrogate the increased expression of ARHGEF15 and ABL2 caused by knockdown of miR-193a (Fig. 4g). Further, we detected relatively lower expression of TGF-β2, TGF-βRIII, ARHGEF15 and ABL2 in both SW1990- and PANC-1-reporter cells co-cultured with irradiated feeder cells than those with untreated ones in vitro (Fig. 4h), and similarly in irradiated tumor tissues than in the untreated ones in vivo (Fig. 4i). These results indicated that miR-193a could destroy intercellular junctions of pancreatic cancer cells, and promote metastasis through inhibiting TGF-β2/TGF-βRIII/ARHGEF15/ABL2 signaling.

### Knockdown of miR-193a or restoration of TGF-β2/TGF-βRIII signalings blocks cancer repopulation and metastasis after radiation

**Fig. 5:**
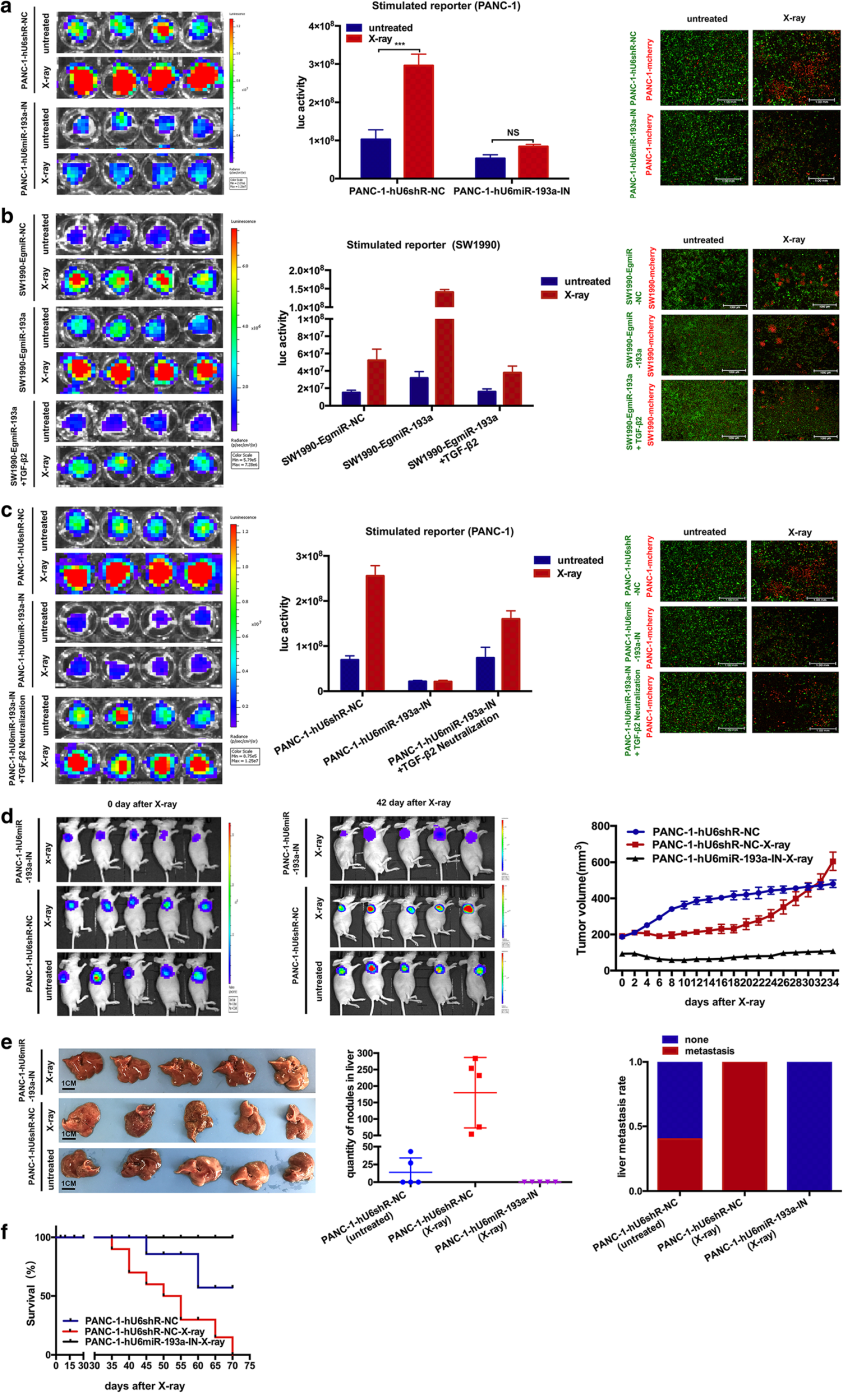
Knockdown of miR-193a or restoration of TGF-β2/TGF-βRIII signaling blocks cancer repopulation and metastasis. **a** Repopulation of PANC-1 reporter cells co-cultured with the corresponding feeder cells (±X-ray). n = 4. *Left*, representative bioluminescence images. *Central*, luciferase activity (photons/s). *Right*, representative fluorescent images. Scale bar, 1 mm. ***p < 0.001. **b** & **c** Repopulation of the stimulated SW1990 and PANC-1 reporter cells co-cultured with the corresponding feeder cells treated with or without TGF-β2 cytokine **b** or neutralization antibody **c**; (±X-ray). n = 4. *Left*, representative bioluminescence images. *Central*, luciferase activity (photons/s). *Right*, representative fluorescent images. Scale bar, 1000 μm. **d** Knockdown of miR-193a blocked pancreatic cancer repopulation in vivo (±X-ray). n = 5. *Left* & *central*, representative bioluminescence images in indicated time. *Right*, tumor growth curve of pancreatic cancer on X-ray radiation. **e** Liver metastasis of the untreated (PANC-1-hU6shR-NC) and the irradiated (PANC-1-hU6miR-193a-IN vs PANC-1-hU6shR-NC) tumor-bearing mice. *Left*, photograph of the dissected liver, n = 5. Scale bar, 1 cm. *Central*, numbers of liver nodules. *Right*, liver metastasis rate. **f** The tumor-bearing mice survival curve on X-ray radiation, *n* = 9

To further explore the necessity of miR-193a in pancreatic cancer repopulation and metastasis, the feeder cells, PANC-1-hU6shR-NC and PANC-1-hU6miR-193a-IN, were treated with or without X-ray, and then co-cultured with their corresponding reporter cells. An obvious increase in the luciferase activity was measured in the stimulated PANC-1-reporter cells co-cultured with the irradiated PANC-1-hU6shR-NC feeder cells in compared to those with untreated PANC-1-hU6shR-NC ones. However, the luciferase activity of the stimulated PANC-1-reporter cells co-cultured with PANC-1-hU6miR-193a-IN feeder cells was almost the same before and after radiation (Fig. 5a, *Left* & *central*). Besides, even under the untreated condition, PANC-1-reporter cells co-cultured with PANC-1-hU6miR-193a-IN feeder cells grew slower than that co-cultured with PANC-1-hU6shR-NC ones. Additionally, in the osculatory co-culture model, PANC-1-mcherry reporter cells only increased in the wells seeded with the irradiated PANC-1-hU6shR-NC feeder cells. No significant difference of PANC-1-mcherry reporter cells was observed when seeded with PANC-1-hU6miR-193a-IN feeder cells between the irradiated and the untreated groups (Fig. 5a, *Right*).

We further observed that the irradiated SW1990 feeder cells could stimulate the growth of co-cultured reporter cells (both SW1990-EgmiR-NC and SW1990-EgmiR-193a), yet the stimulated SW1990-EgmiR-193a reporter cells obviously grew faster than the SW1990-EgmiR-NC ones (Additional file 12: Figure S7). However, the irradiated PANC-1 feeder cells could only stimulate the growth of the co-cultured PANC-1-hU6shR-NC reporter cells, yet knockdown of miR-193a in PANC-1 reporter cells blocked its stimulation from the irradiated PANC-1 feeder cells. Besides, even co-cultured with the irradiated PANC-1 feeder cells, the growth of PANC-1-hU6miR-193a-IN reporter cells was slower than that of PANC-1-hU6shR-NC reporter cells co-cultured with the untreated PANC-1 feeder cells. Collectively, miR-193a was indispensable for pancreatic cancer repopulation in vitro*.*

Ulteriorly, in gain- and loss-of-function assay, higher miR-193a in the feeder cells either induced by ectopic expression or radiation could promote the growth of SW1990-reporter cells, leading to the living pancreatic cancer cell repopulation. However, knockdown of miR-193a in PANC-1 feeder cells inhibited the repopulation after radiation. Additionally, restoration of TGF-β2 could inhibit the growth of SW1990 reporter cells co-cultured with the untreated SW1990-EgmiR-193a feeder cells, more than just inhibited the repopulation (Fig. 5b). Yet, neutralization of TGF-β2 in the co-culture system with PANC-1-hU6miR-193a-IN feeder cells led to restoration of the growth in PANC-1-reporter cells. Even for PANC-1-reporter cells co-cultured with the untreated PANC-1-hU6miR-193a-IN ones, inhibition of TGF-β2 could make its growth resumption (Fig. 5c). These results indicated that TGF-β2 could counteract the effects of pancreatic cancer cell proliferation and repopulation induced by miR-193a overexpression.

We further explored whether miR-193a was necessary in pancreatic cancer repopulation and metastasis in vivo. PANC-1-hU6miR-193a-IN and PANC-1-hU6shR-NC cancer cells were subcutaneously injected into nude mice, and administered X-ray radiation. About 4 days after radiation, the PANC-1-hU6shR-NC-cell-loaded tumor became to shrink slowly, and the tumor volume reduced to the minimum (~192mm^3^ at average) about one week later. Then the irradiated tumor recovered and began to grow up slowly. About 3 weeks later, the growth pace of the irradiated tumor was suddenly accelerated and much faster than before. However, the untreated tumor grew up steadily without shrinkage and accelerated expansion (Fig. 5d). Nevertheless, the PANC-1-hU6miR-193a-IN cell-loaded tumor also shrank after radiation, yet kept at a near-constant volume in the following one month without accelerated expansion (~107mm^3^ at average), even similar to the volume before radiation (~94mm^3^ at average). The results indicated that knockdown of miR-193a blocked pancreatic cancer repopulation in vivo*.*

Moreover, the mice with PANC-1-hU6shR-NC-cell-loaded tumor was found to present a higher rate of spontaneous liver metastasis after radiation than the untreated group. The rate of liver metastasis even achieved 100% in the irradiated group (Fig. 5e). However, the irradiated mice with PANC-1-hU6miR-193a-IN-cell-loaded tumor showed no metastatic nodule. The mice burdened with PANC-1-hU6miR-193a-IN cell lived longer than the other two group mice after X-ray radiation (Fig. 5f). These data demonstrated that knockdown of miR-193a or restoration of TGF-β2/TGF-βRIII signalings in pancreatic cancer cells could inhibit cancer repopulation and metastasis after radiation, and prolong the survival.

### miR-193a antagonist suppresses cancer repopulation and progression in PDX model

**Fig. 6:**
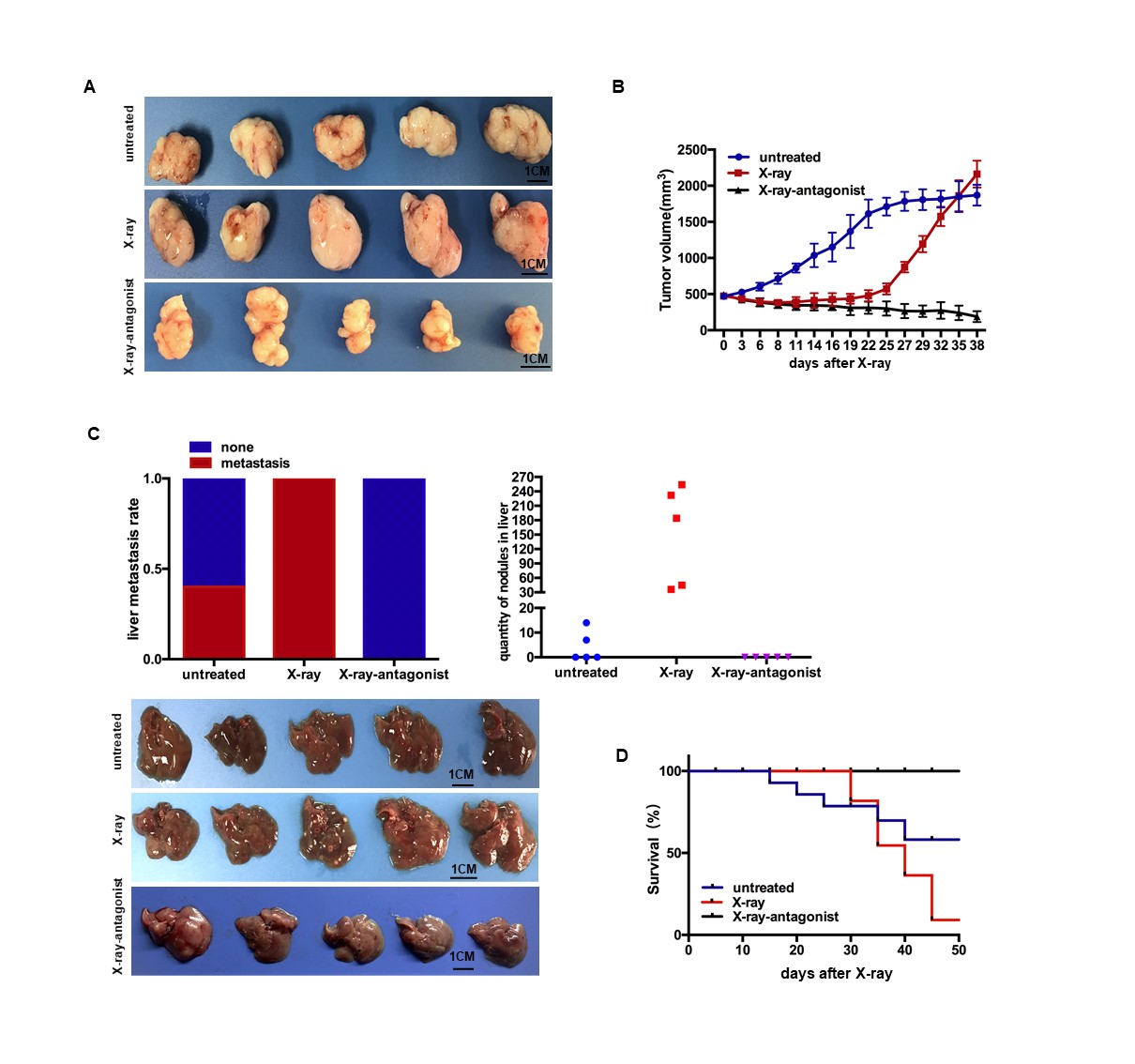
Targeting miR-193a suppresses cancer repopulation and metastasis in PDX model. **a** Representative images of the dissected tumor in PDX mice. n = 5. Scale bar, 1 cm. **b** PDX tumor growth curve on X-ray radiation with miR-193a antagonist treatment. **c** Liver metastasis of PDX. *Upper left*, liver metastasis rate. *Upper right*, numbers of liver nodules. *Lower*, photograph of the dissected liver, n = 5. Scale bar, 1 cm. **d** PDX mice survival curve on X-ray radiation with miR-193a antagonist treatment, *n* = 11

Patient-derived xenograft (PDX) models of pancreatic cancer were further established, and administered X-ray radiation when tumor grew to ~500mm^3^. Meanwhile, another group of the irradiated PDX mice were treated with miR-193a antagonist. 3 days after radiation, the tumor volume of the irradiated PDX group became to shrink slowly. One week later, the tumor of the irradiated PDX recovered and began to grow up slowly. About 3 weeks after radiation, the growth of the irradiated PDX was suddenly accelerated (Fig. 6a *middle* & 6B). However, the untreated PDX grew up steadily without shrinkage and accelerated expansion (Fig. 6a *Upper* & 6b). Notably, the irradiated PDX group with miR-193a antagonist treatment shrank slowly after radiation, and failed to recover (Fig. 6a *Lower* & 6b). At the last observation time-point, the tumor volume of the irradiated PDX with miR-193a antagonist treatment (190mm^3^ at average) was far less than those of the untreated (1869mm^3^ at average) and the irradiated (2161mm^3^ at average) group, and even smaller than its volume before radiation (493mm^3^ at average) (Fig. 6a & 6b). These results showed that miR-193a antagonist treatment could block pancreatic cancer repopulation in PDX model.

In addition, there appeared a higher rate of spontaneous liver metastasis in PDX with radiation than the untreated group, and even reached 100% of liver metastasis. However, no tumor metastasis appeared in the irradiated PDX group with miR-193a antagonist treatment (Fig. 6c, *Upper left* & *Lower*). Of note, the numbers of the metastatic nodules in the irradiated PDX group with miR-193a antagonist treatment (0 at average) were much less than that of the only irradiated one (> 100 at average, 36~ 254 in range), and even less than that of the untreated one (< 5 at average) (Fig. 6c, *Upper right*). Moreover, the irradiated PDX group with miR-193a antagonist treatment lived much longer than the only irradiated or the untreated one (Fig. 6d). These data indicated that miR-193a antagonist in pancreatic cancer could repress metastasis after radiation, and improve the survival. In other words, miR-193a could serve as a potential therapeutic target to resensitize cancer cell to radiation, and abrogate repopulation after radiation in pancreatic cancer.

## Discussion

Pancreatic cancer is known to be usually diagnosed at a late stage, and patients often lose surgery opportunity. Especially for locally advanced pancreatic cancer, radiotherapy is usually preferred [34]. Unfortunately, pancreatic cancer is still apt to repopulate and relapse even after radiotherapy. Repopulation is regarded as a major cause of tumor relapse after radiotherapy [5]. It is urgent to understand the underlying mechanisms and explore novel targets for intervention of pancreatic cancer repopulation.

**Fig. 7:**
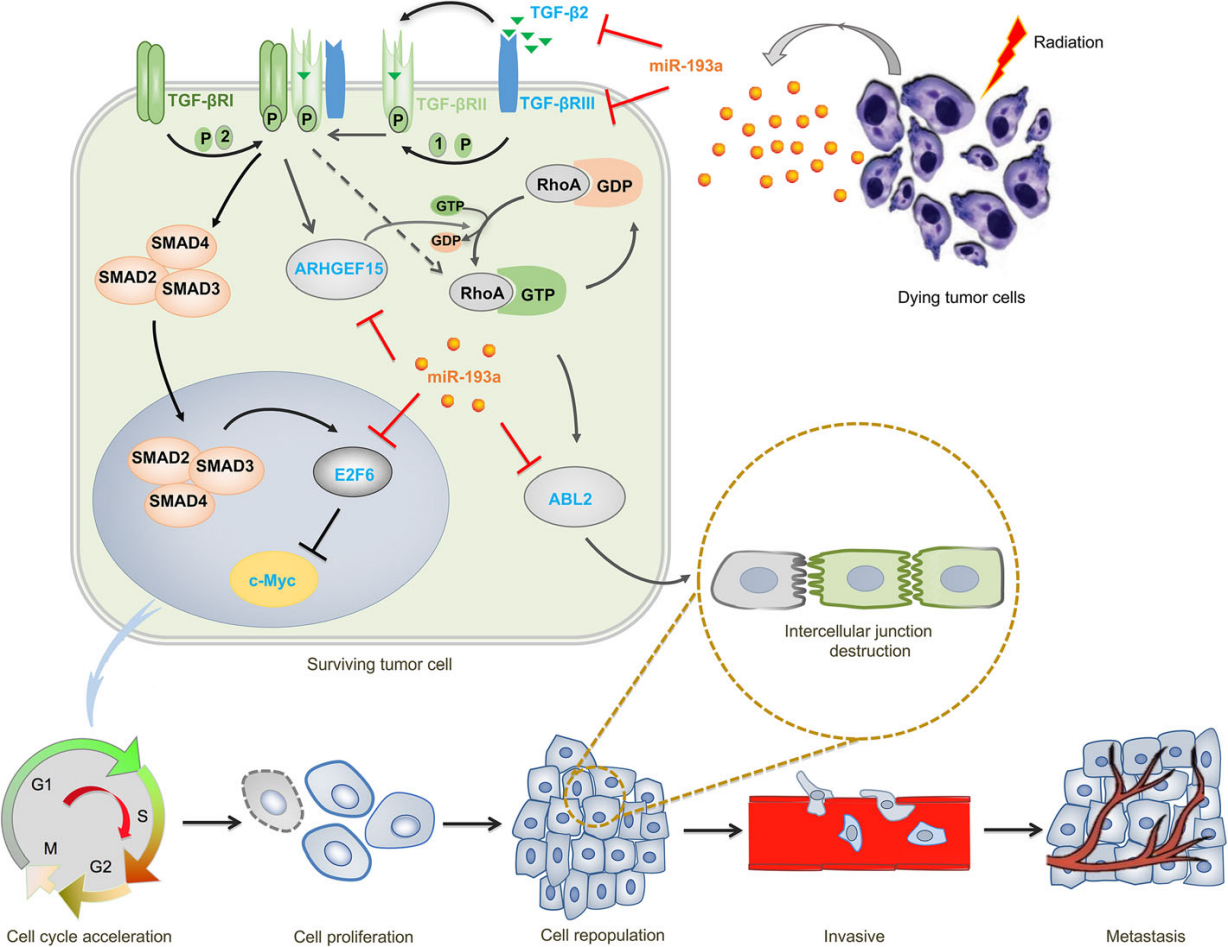
miR-193a from the irradiated dying cells stimulates pancreatic cancer cell proliferation, repopulation and metastasis

As known, after exposure to the ionizing radiation, the overwhelming majority of tumor cells are killed, the minority of surviving tumor cells can escape and repopulate subsequently [35]. The underlying mechanisms of tumor repopulation have been revealed in recent years. It was considered that cancer stem cells could proliferate residual tumor cells into accelerated repopulation via PGE_2_ in bladder cancer [6]. Radiation was found to upregulate tumor angiogenesis through activating HIF-1 [36] and modulating caspase-3 [37]. The integrity of endothelial cells was also considered to involve in cancer repopulation [38]. Our previous studies reported that the dying cells from chemoradiation could stimulate the surviving cancer cells into repopulation by activating “phoenix rising” signaling and the succedent pathways [7–10]. In this study, we further revealed that miR-193a promoted pancreatic cancer repopulation and metastasis through modulating TGF-β2/TGF-βRIII signaling (Fig. 7). As shown, miR-193a was found to be highly expressed in the irradiated pancreatic cancer cells, and be transferred into the surviving pancreatic cancer cells. Then on the one hand, the elevated miR-193a in the surviving cells accelerated cell cycle through inhibiting TGF-β2/TGF-βRIII/SMADs/E2F6/c-Myc signaling, and led to cell proliferation and repopulation. On the other hand, miR-193a destroyed normal intercellular junctions through repressing TGF-β2/TGF-βRIII/ARHGEF15/ABL2 signaling, and caused the repopulated cancer cells to deviate from the primary sites and migrate for metastasis.

In terms of mechanisms, a complex network of cellular responses was evoked to the ionizing radiation damage [39]. At least, four canonical signalings including PI3K/AKT, MAPK/ERK, NF-κB and TGF-β were involved [12]. Particularly, through interplaying with the key components in radiation-related signals, miRNAs might play a critical role in the modulation of radiation responses [12]. As known, one miRNA could simultaneously regulate multiple genes through mRNA destabilization or translational inhibition, leading to global influences on diverse biological processes of cancer cells. It was reported that miR-21 could modulate cell cycle, and inhibition of miR-21 could reduce G2/M accumulation induced by ionizing radiation in glioblastoma [40, 41]. miR-17-92 cluster could regulate collagen synthesis through TGF-β pathway [17]. We herein found that miR-193a, which increased in the irradiated cells, could accelerate cell cycle and destroy intercellular junctions to promote metastasis by modulating TGF-β2/TGF-βRIII signalings.

However, some controversies about TGF-β2 in cancer remain unsolved. Some researches insisted that TGF-β2 played a growth-suppressive role in cancer [42], and contributed to discriminate the restrictive or the permissive metastasis microenvironment [23]. Yet, others thought that higher TGF-β2 expression assumed greater occurrence rate of invasion and metastasis in pancreatic cancer [43]. It was found that serous TGF-β2 increased in pancreatic cancer patients, and was correlated with poor survival [44]. In our study, we found that TGF-β2/TGF-βRIII signaling was repressed in pancreatic cancer repopulation and metastasis. Restoration of TGF-β2 could suppress pancreatic cancer repopulation, and vice versa in vitro. We thought TGF-β2 played suppressive roles in pancreatic cancer repopulation and secondary metastasis.

Remarkably, TGF-βRIII, an indispensable receptor for TGF-β2, was found to suppress tumorigenesis in prostate cancer [45], breast cancer [46] and ovarian cancer [47]. Knockdown of TGF-βRIII in non-metastatic NMuMG cells resulted in E-cadherin decrease and promoted cells to form more invasive structure in mice [48]. Some studies also verified that TGF-βRIII could induce cell growth-arrest [23], and TGF-βRIII expression decreased in pancreatic cancer. Loss of TGF-βRIII was companied with advanced tumor grade, invasion and metastasis [49]. Hereinabove, we also found that TGF-βRIII was down-regulated during the process of pancreatic cancer cell proliferation, repopulation and metastasis after radiation. These findings supported that TGF-βRIII also made suppressive effects on pancreatic cancer repopulation and succedent metastasis after radiation.

Furthermore, TGF-β2 shows low affinity to TGF-βRII and TGF-βRI and requires TGF-βRIII to assist its presentation [21]. In our study, besides miR-193a could regulate TGF-β2 expression, miR-193a was also found to directly target TGF-βRIII. The miR-193a-mediated loss-of-function for TGF-βRIII made TGF-β2 unable to combine with TGF-βRII, further inactivating TGF-β2 signaling.

In addition, we found that pancreatic cancer cells or the loaded-tumors of mice with low-level miR-193a failed to undergo repopulation after radiation. The PDX mice with miR-193a antagonist treatment effectively inhibited tumor repopulation and metastasis, and exhibited an improved prognosis after radiotherapy. Therefore, the expression level of miR-193a in pancreatic cancer tissues might contribute to screen the beneficial patients for radiotherapy. Whilst, miR-193a intervention might help to improve pancreatic cancer radiotherapy. Moreover, restoration of TGF-β2 signaling with cytokine could block miR-193a-induced repopulation in pancreatic cancer. Thereby, targeting TGF-β2 signalings could also be a promising therapeutic strategy for pancreatic cancer repopulation. Virtually, some drugs targeting TGF-β2, such as Trabedersen (AP12009), Belagen-pumatucel-L (Lucanix), Lerdelimumab (CAT-152), have been developed in preclinical or clinical trials [50, 51]. Notably, as an indispensable receptor for TGF-β2, TGF-βRIII was also a potential drug target. Disitertide (P144), an inhibitor of TGF-βRIII, has been developed and entered phase II clinical trial [50]. Taken together, our findings indicated that miR-193a antagonist could effectively block pancreatic cancer repopulation, reduce spontaneous liver metastasis and prolong the survival through modulating TGF-β2/TGF-βRIII signalings. miR-193a might be a potential therapeutic target for pancreatic cancer repopulation and metastasis after chemoradiation treatment.

## Conclusion

Our findings elucidated a potential mechanism of pancreatic cancer repopulation and metastasis after chemoradiation treatment. We herein identified that the elevated miR-193a from the irradiated dying pancreatic cancer cells could promote the surviving cancer cell repopulation and metastasis through modulating TGF-β2/TGF-βRIII signaling. Furthermore, we presented a potential therapy option for pancreatic cancer. The treatment of radiation in combination with miR-193a antagonist could effectively inhibit pancreatic cancer cell repopulation and metastasis.

## Additional files

Additional file 1: Table S1. PCR primers used in this study. **Table S2.** Synthesized oligonucleotides in this study. (DOCX 17 kb)

Additional file 2: Figure S1. Schematic physical maps of the constructs concerning miR-193a targets. The 3’-UTR of predicted miR-193a binding site in TGF-β2, TGF-βRIII, ABL2, E2F6 and ARHGEF15, and their putative counterparts (WTs and MTs) were inserted into the reporter plasmid pmirGLO. The recombinants were accordingly constructed. Their physical maps of these constructs were schematically shown. (TIFF 1264 kb)

Additional file 3: Figure S2. Schematic physical maps of the constructs for developing stable cells. The amplified pri-miR-193a was cloned into the expression plasmid of pLEX-EgmiR-CvG2L, and resulted in pLEX-EgmiR-193a/CvG2L. The synthetized miR-193a inhibitor (miR-193a-IN) was cloned into the vector pLV-hU6shRNA/CvG2L, and resulted in pLV-hU6miR-193a-IN/CvG2L. The schematic physical maps of these constructs were accordingly shown. (TIFF 625 kb)

Additional file 4: Figure S3. The basic characteristics of pancreatic epithelial cell (HPDE6-C7) and cancer cells (PANC-1, SW1990 and AsPC-1). (A) The basal miR-193a expression was assessed by RT-qPCR assay. *p < 0.05, **p < 0.01. *n* = 3. (B) SMAD4 protein expression was tested by western blot. (TIFF 490 kb)

Additional file 5: Figure S4. Pancreatic cancer repopulation model. (A) Schematic diagram of pancreatic cancer repopulation in vitro models. *Left*, the detached co-cultured system; *Right*, the osculatory co-cultured system. Green cells presented luciferase /GFP-labeled feeder cells, and red cells were mcherry-labeled reporter cells. The ratio of feeder and reporter cells was 100:1. (B&C) *R*epopulation of SW1990- (B) and PANC-1-(C) reporter cells co-cultured with corresponding feeder cells (no feeder cells, SW1990- and PANC-1-feeder cells; ±X-ray). *n* = 4. *Left*, representative bioluminescence images. *Central*, luciferase activity (photons/s). *Right*, representative fluorescent images. Scale bar, 1 mm. **p < 0.01. (D) Schematic diagram of pancreatic cancer repopulation in vivo model. (E) Representative bioluminescence images in indicated time. (TIFF 3615 kb)

Additional file 6: Figure S5. The basic characteristics of the stable cells. (A) miR-193a expression changes in stable cells were assessed by RT-qPCR assay. *Left*, SW1990-EgmiR-193a vs SW1990-EgmiR-NC; *Right*, PANC-1-hU6shR-NC vs PANC-1-hU6miR-193a-IN. ***p < 0.001. n = 3. (B) Cell cycle of stimulated reporter cells co-cultured with corresponding untreated (*Left*) and irradiated feeder cells (*Right*) were analyzed by ModFit LT software. *Upper*, SW1990 cells; *Lower*, PANC-1 cells. (TIFF 1573 kb)

Additional file 11: Figure S6. ARHGEF15 and ABL2 were the target genes of miR-193a. (A) Schematic diagrams of the predicted miR-193a-binding sites in 3’-UTR of ARHGEF15 and ABL2. (B) Relative luciferase activity on 3’-UTR, WT and MT counterparts were measured. ***p < 0.001. n = 3. (C) Western blot for detecting ARHGEF15 and ABL2 proteins in SW1990 cells transfected with miR-193a angomir and AsPC-1 cells transfected with miR-193a antagomir. (TIFF 762 kb)

Additional file 12: Figure S7. Repopulation of SW1990-EgmiR-NC vs SW1990-EgmiR-193a (A), and PANC-1-hU6shR-NC vs -PANC-1-hU6miR-193a-IN reporter cells (B) co-cultured with corresponding SW1990 and PANC-1 feeder cells. All feeder cells were irradiated with lethal dose (10Gy) except the top line and fourth line. n = 4. The luciferase activity was shown as photons/s. **p < 0.01, ***p < 0.001. (C) Presentative bioluminescence images. (TIFF 1898 kb)

## Abbreviations

ABL2: V-abl Abelson murine leukemia viral oncogene homolog 2
ARHGEF15: Rho guanine nucleotide exchange factor 15
E2F6: elongation factor 2 transcription factor 6
miR-193a: microRNA-193a
miRNA: microRNA
PDX: patient-derived tumor xenograft
PGE2: prostaglandin E2
TGF-β2: transforming growth factor beta 2
TGF-βRIII: transforming growth factor beta receptor type 3
