# Correction to: microRNA-193a stimulates pancreatic cancer cell repopulation and metastasis through modulating TGF-β2/TGF-βRIII signalings

**DOI:** 10.1186/s13046-021-02180-6

**Published:** 2021-12-31

**Authors:** Chi Fang, Chen-yun Dai, Zhu Mei, Ming-jie Jiang, Dian-na Gu, Qian Huang, Ling Tian

**Affiliations:** 1 Institute of Translational Medicine, Science bldg. Rm 205, Shanghai General Hospital, Shanghai Jiao Tong University School of Medicine, New Songjiang Rd No.650, Songjiang District, Shanghai, 201620 China; 2 Shanghai Key Laboratory of Pancreatic Diseases, Shanghai General Hospital, Shanghai Jiao Tong University School of Medicine, Shanghai, China; 3 Department of Gastroenterology, Shanghai General Hospital, Shanghai Jiao Tong University School of Medicine, Shanghai, China; 4 The Comprehensive Cancer Center, Shanghai General Hospital, Shanghai Jiao Tong University School of Medicine, Shanghai, China

**Correction to: J Exp Clin Cancer Res 37, 25 (2018)**

**https://doi.org/10.1186/s13046-018-0697-3**

Following publication of the original article [1], the authors identified minor errors in Fig. 6 specifically:

- Figure 6c: incorrect image was used for the lower (X-ray-antagonist) panel.

**Fig. 6:**
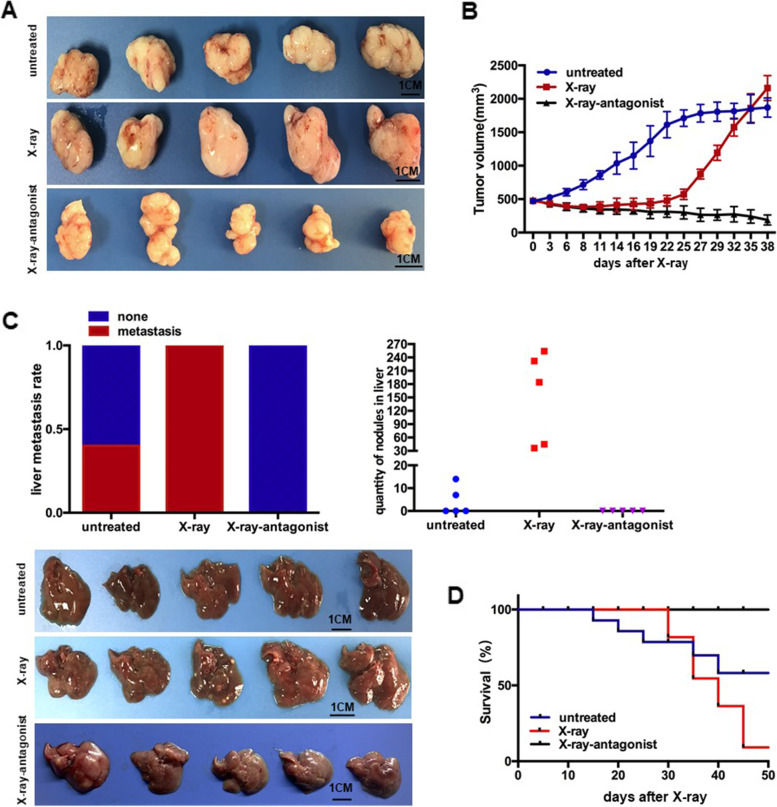
Targeting miR-193a suppresses cancer repopulation and metastasis in PDX model. **a** Representative images of the dissected tumor in PDX mice. *n* = 5. Scale bar, 1 cm. **b** PDX tumor growth curve on X-ray radiation with miR-193a antagonist treatment. **c** Liver metastasis of PDX. *Upper left*, liver metastasis rate. *Upper right*, numbers of liver nodules. *Lower*, photograph of the dissected liver, *n* = 5. Scale bar, 1 cm. **d** PDX mice survival curve on X-ray radiation with miR-193a antagonist treatment, *n* = 11

The authors provided the Journal with the original data files. The corrected figure is provided here. The correction does not have any effect on the results or conclusions of the paper. The original article has been corrected.
