# Supplementary material for: microRNA-193a stimulates pancreatic cancer cell repopulation and metastasis through modulating TGF-β2/TGF-βRIII signalings
Source: J Exp Clin Cancer Res. 2018 Feb 13;37:25. doi: 10.1186/s13046-018-0697-3 (PMC5809917; doi:10.1186/s13046-018-0697-3)
Supplement: Supplementary file 5 — PCR primers used in this study. Table S2. Synthesized oligonucleotides in this study. (DOCX 17 kb) [file 13046_2018_697_MOESM1_ESM.docx]

**Table S1. Sequences for primers used in this study**

| **Primers** | **Sequences** |
| --- | --- |
| TGF-β2 (Forward) | 5’-CGAGCTCGGAAAAGTGGCAAGACCAAA-3’ |
| TGF-β2 (Reverse) | 5’ -GCTCTAGACAAAGCACAAGTAGGGCAAA-3’ |
| TGF-βRIII (Forward) | 5’ -CGAGCTC GAGCACATTTTGTTGGGTGA-3’ |
| TGF-βRIII (Reverse) | 5’ -GCTCTAGAAACAGGGGTGTTCAAACTGG-3’ |
| ABL2 (Forward) | 5’- CGAGCTCCGGGGCCTAGCTGTGTATAA-3’ |
| ABL2 (Reverse) | 5’- GCTCTAGA TACACGGCAACAATTCCAAA-3’ |
| E2F6 (Forward) | 5’-CGAGCTCTCGTGGAGAATTACGCATCA-3’ |
| E2F6 (Reverse) | 5’-GCTCTAGAGGGAAGGGTAGAGTCCACAA-3’ |
| ARHGEF15 (Forward) | 5’-CGAGCT CCTGAGCTTTGGGTCTCTTGG-3’ |
| ARHGEF15 (Reverse) | 5’-GCTCTAGA GTTTGGCCCAGTGTTTCTGT-3’ |
| miR-193a (Forward) | 5’-CAGGATCC AGTTTCTCGGCGCATAACTCT-3’ |
| miR-193a (Reverse) | 5’-GAAGCTAGCAGGGGCAATTCTAGAAGTCAG-3’ |

**Table S2.** **Synthesized oligonucleotides in this study**

| **Name** | **Sequence** |
| --- | --- |
| ^*^BS_TGF-β2_-WT1 | 5’-ACGAGCTCATGTTTCTTTTAGCTGGCCAGTATCTAGACG -3’ |
| BS_TGF-β2_-WT2 | 5’-CGTCTAGATACTGGCCAGCTAAAAGAAACATGAGCTCGT -3’ |
| BS_TGF-β2_-MT1 | 5’-ACGAGCTCATGTTTCTTTTAGCTGACGTCAATCTAGACG -3’ |
| BS_TGF-β2_-MT2 | 5’-CGTCTAGATTGACGTCAGCTAAAAGAAACATGAGCTCGT-3’ |
| BS_TGF-βRIII_-WT1 | 5’- ACGAGCTCCCTTAAAATCCCTGTGGCCAGTTTCTAGACG-3’ |
| BS_TGF-βRIII_-WT2 | 5-CGTCTAGAAACTGGCCACAGGGATTTTAAGGGAGCTCGT -3’ |
| BS_TGF-βRIII_-MT1 | 5’-ACGAGCTCCCTTAAAATCCCTGTGACGTCATTCTAGACG-3’ |
| BS_TGF-βRIII_-MT2 | 5’-CGTCTAGAATGACGTCACAGGGATTTTAAGGGAGCTCGT -3’ |
| BS_ABL2_-WT1 | 5’-ACGAGCTCACAATTTGTGATCTGGCCAGTTCGAGGGAGCAGT CTGTTGCCAGTAATCTAGAGC -3’ |
| BS_ABL2_-WT2 | 5’-GCTCTAGATTACTGGCAACAGACTGCTCCCTCGAACTGGCCA GATCACAAATTGTGAGCTCGT -3’ |
| BS_ABL2_-MT1 | 5’-AGGAGCTCACAATTTGTGATCTGACGTCATCGAGGGAGCAGT CTGTTGACGTCAATCTAGAGC -3’ |
| BS_ABL2_-MT2 | 5’-GCTCTAGATTGACGTCAACAGACTGCTCCCTCGATGACGTCA GATCACAAATTGTGAGCTCCT -3’ |
| BS_E2F6_-WT1 | 5’-ACGAGCTCCAGTAGCGGCATCATGGCCAGTATCTAGACG-3’ |
| BS_E2F6_-WT2 | 5’-CGTCTAGATACTGGCCATGATGCCGCTACTGGAGCTCGT-3’ |
| BS_E2F6_-MT1 | 5’-ACGAGCTCCAGTAGCGGCATCATGACGTCAATCTAGACG -3’ |
| BS_E2F6_-MT2 | 5’-CGTCTAGATTGACGTCATGATGCCGCTACTGGAGCTCGT-3’ |
| BS_ARHGEF15_-WT1 | 5’-ACGAGCTCCAAGGGACTGAAGATGGCCAGTATCTAGACG -3’ |
| BS_ARHGEF15_-WT2 | 5’- CGTCTAGATACTGGCCATCTTCAGTCCCTTGGAGCTCGT -3’ |
| BS_ARHGEF15_-MT1 | 5’-ACGAGCTCCAAGGGACTGAAGATGACGTCAATCTAGACG -3’ |
| BS_ARHGEF15_-MT2 | 5’- GCTCTAGATTGACGTCATCTTCAGTCCCTTGGAGCTCGA -3’ |
| miR-193a-IN | 5’-GGACTAGTGGCGCTAGGATCATCAACACTGGGACTTTATCTG TAGGCCAGTTCAAGTATTCTGGTCACAGAATACAACACTGGGAC TTTATCTGTAGGCCAGTTCAAGATGATCCTAGCGCCACCTTTTTTGCTAGCTAG-3’ |

*BS: predicted miR-193a binding sequence.
