# Supplementary figures and images for: microRNA-193a stimulates pancreatic cancer cell repopulation and metastasis through modulating TGF-β2/TGF-βRIII signalings
Source: J Exp Clin Cancer Res. 2018 Feb 13;37:25. doi: 10.1186/s13046-018-0697-3 (PMC5809917; doi:10.1186/s13046-018-0697-3)

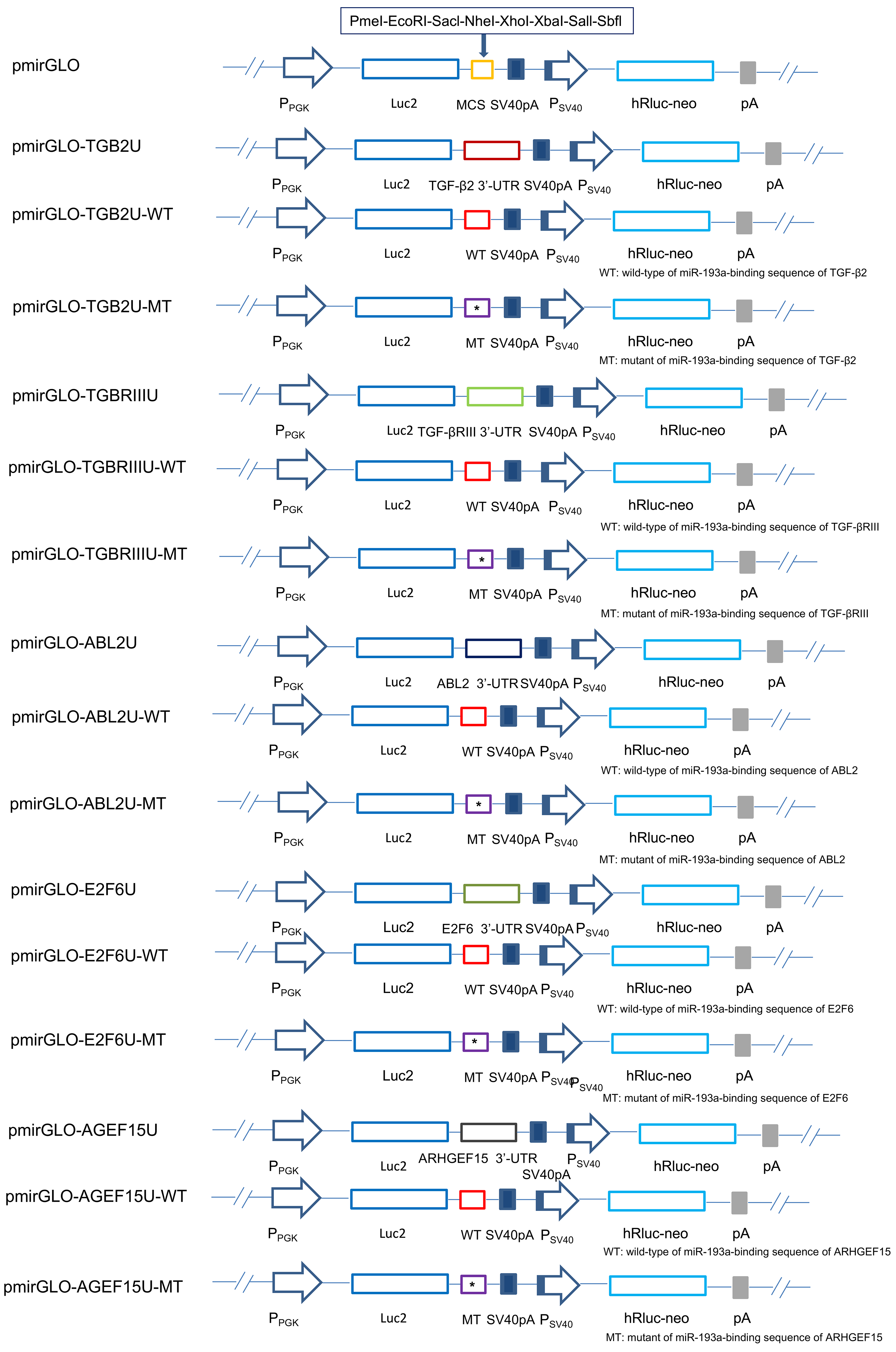

Supplement: Supplementary file 6 — Schematic physical maps of the constructs concerning miR-193a targets. The 3’-UTR of predicted miR-193a binding site in TGF-β2, TGF-βRIII, ABL2, E2F6 and ARHGEF15, and their putative counterparts (WTs and MTs) were inserted into the reporter plasmid pmirGLO. The recombinants were accordingly constructed. Their physical maps of these constructs were schematically shown. (TIFF 1264 kb) [file 13046_2018_697_MOESM2_ESM.tif]

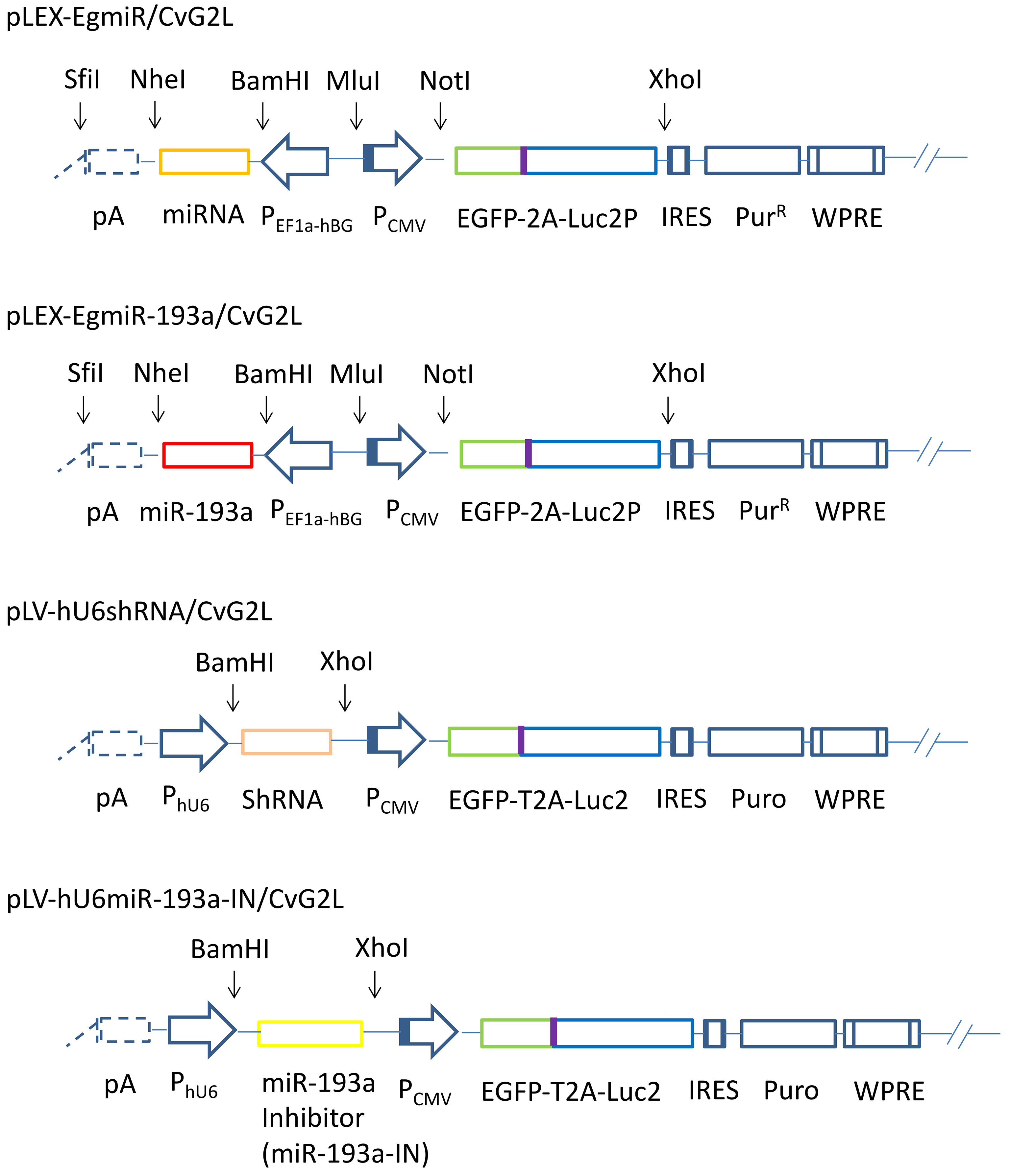

Supplement: Supplementary file 7 — Schematic physical maps of the constructs for developing stable cells. The amplified pri-miR-193a was cloned into the expression plasmid of pLEX-EgmiR-CvG2L, and resulted in pLEX-EgmiR-193a/CvG2L. The synthetized miR-193a inhibitor (miR-193a-IN) was cloned into the vector pLV-hU6shRNA/CvG2L, and resulted in pLV-hU6miR-193a-IN/CvG2L. The schematic physical maps of these constructs were accordingly shown. (TIFF 625 kb) [file 13046_2018_697_MOESM3_ESM.tif]

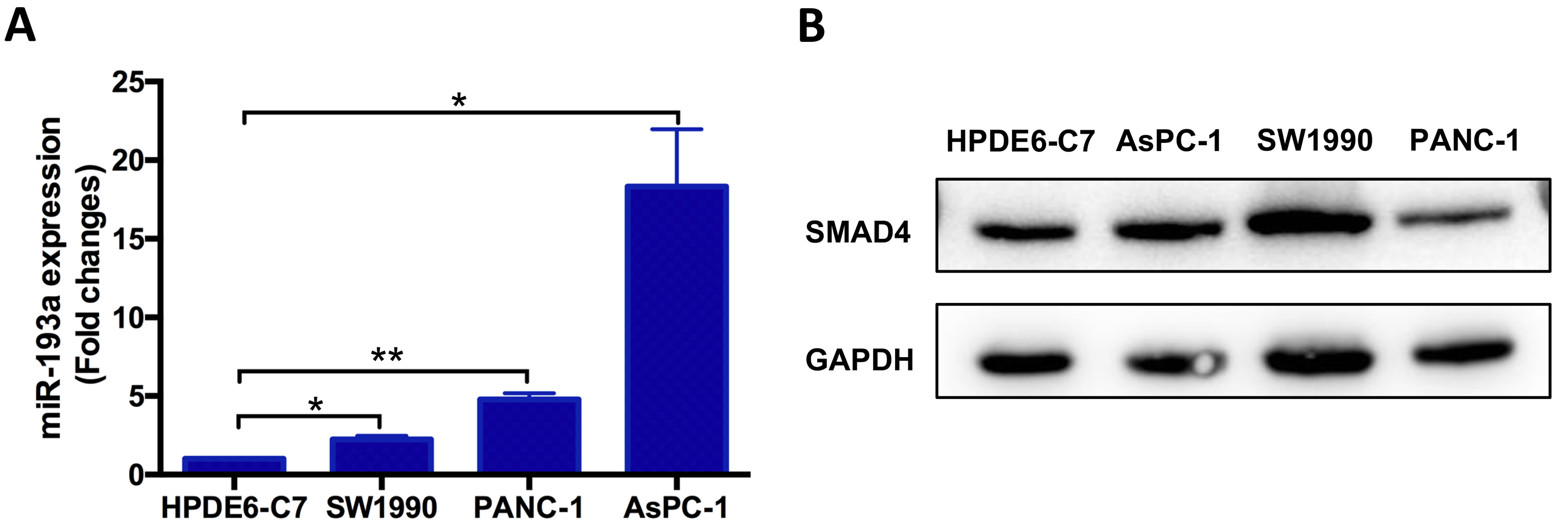

Supplement: Supplementary file 8 — The basic characteristics of pancreatic epithelial cell (HPDE6-C7) and cancer cells (PANC-1, SW1990 and AsPC-1). (A) The basal miR-193a expression was assessed by RT-qPCR assay. *p < 0.05, **p < 0.01. n = 3. (B) SMAD4 protein expression was tested by western blot. (TIFF 490 kb) [file 13046_2018_697_MOESM4_ESM.tif]

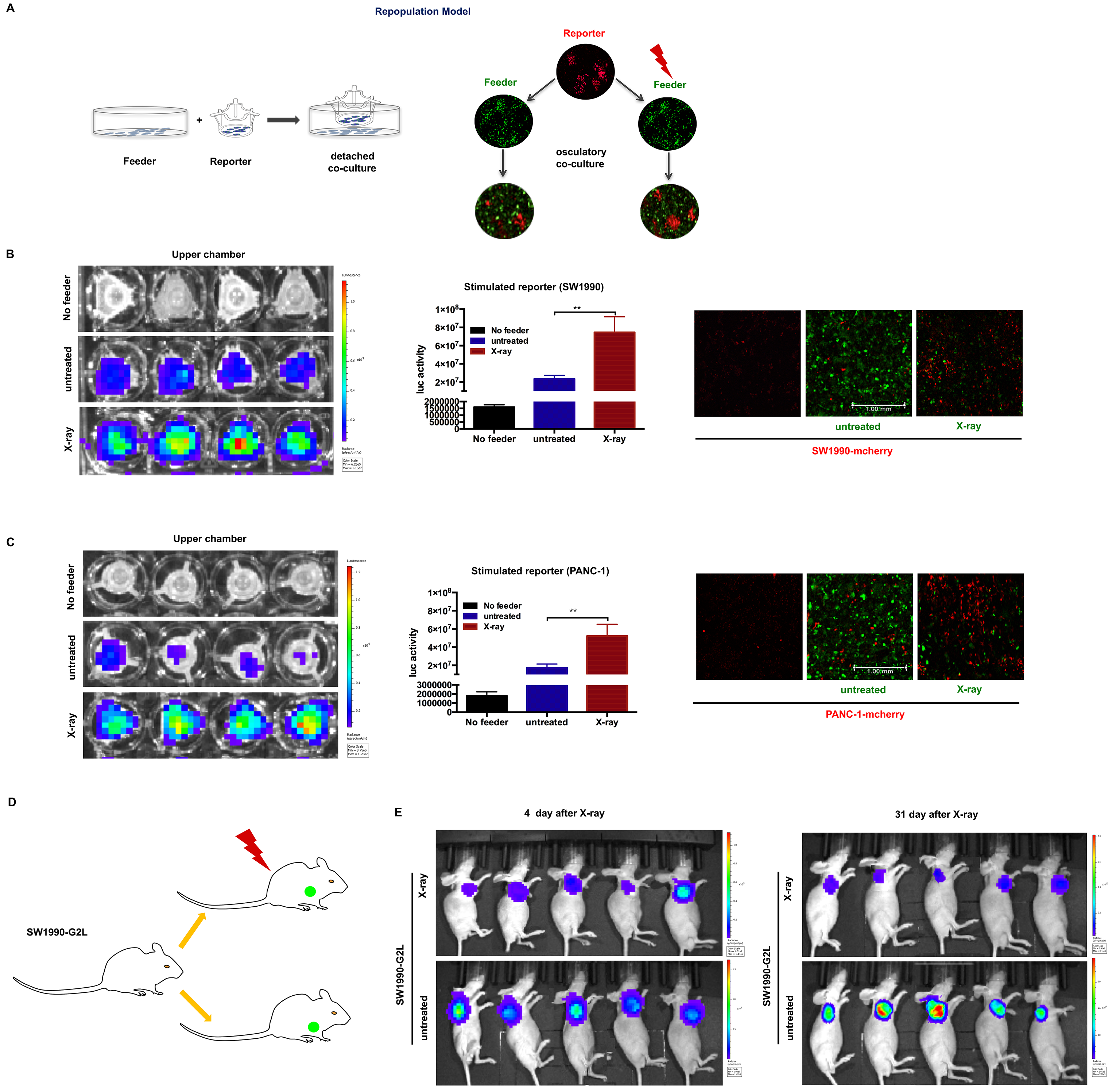

Supplement: Supplementary file 9 — Pancreatic cancer repopulation model. (A) Schematic diagram of pancreatic cancer repopulation in vitro models. Left, the detached co-cultured system; Right, the osculatory co-cultured system. Green cells presented luciferase /GFP-labeled feeder cells, and red cells were mcherry-labeled reporter cells. The ratio of feeder and reporter cells was 100:1. (B&C) Repopulation of SW1990- (B) and PANC-1-(C) reporter cells co-cultured with corresponding feeder cells (no feeder cells, SW1990- and PANC-1-feeder cells; ±X-ray). n = 4. Left, representative bioluminescence images. Central, luciferase activity (photons/s). Right, representative fluorescent images. Scale bar, 1 mm. **p < 0.01. (D) Schematic diagram of pancreatic cancer repopulation in vivo model. (E) Representative bioluminescence images in indicated time. (TIFF 3615 kb) [file 13046_2018_697_MOESM5_ESM.tif]

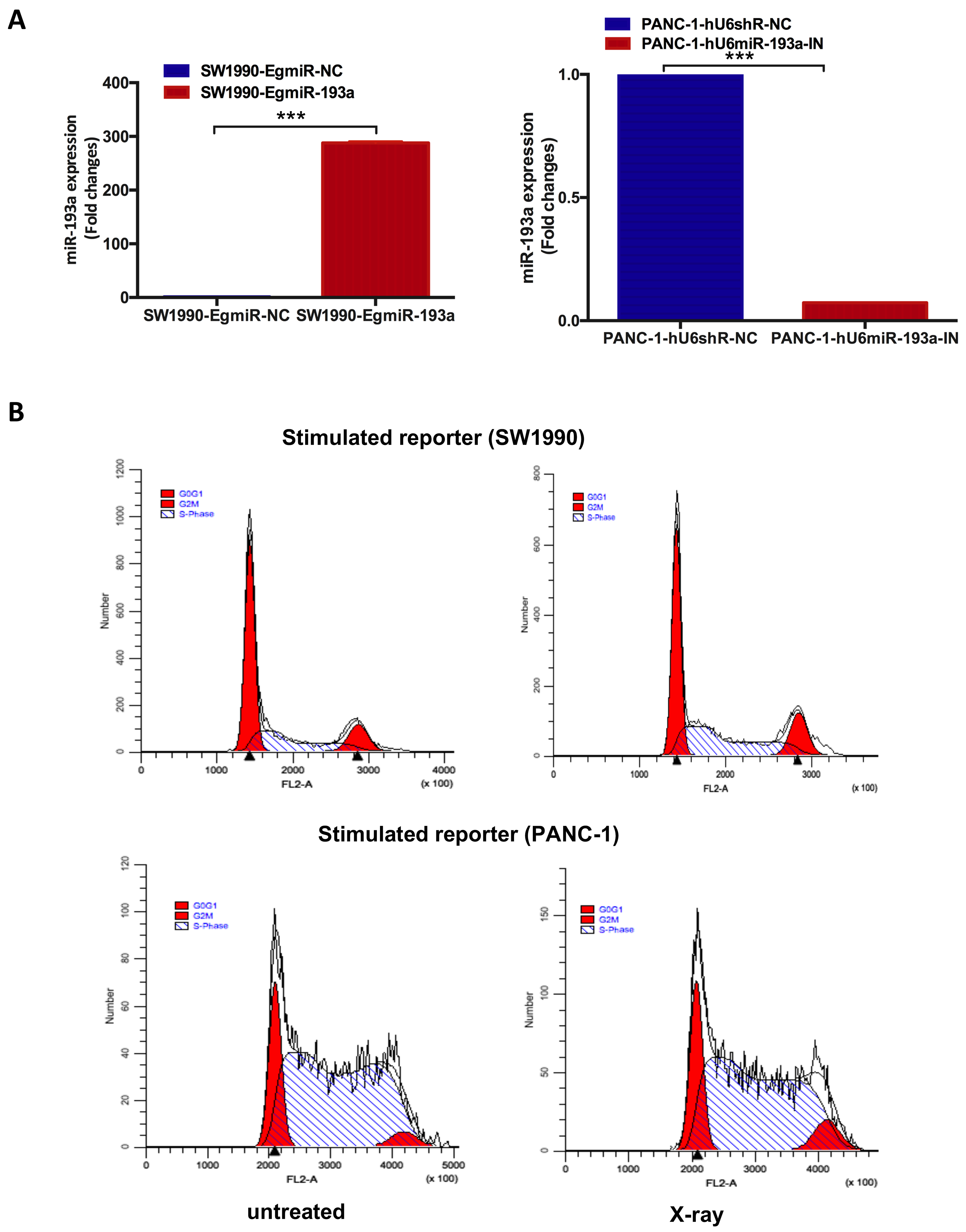

Supplement: Supplementary file 10 — The basic characteristics of the stable cells. (A) miR-193a expression changes in stable cells were assessed by RT-qPCR assay. Left, SW1990-EgmiR-193a vs SW1990-EgmiR-NC; Right, PANC-1-hU6shR-NC vs PANC-1-hU6miR-193a-IN. ***p < 0.001. n = 3. (B) Cell cycle of stimulated reporter cells co-cultured with corresponding untreated (Left) and irradiated feeder cells (Right) were analyzed by ModFit LT software. Upper, SW1990 cells; Lower, PANC-1 cells. (TIFF 1573 kb) [file 13046_2018_697_MOESM6_ESM.tif]

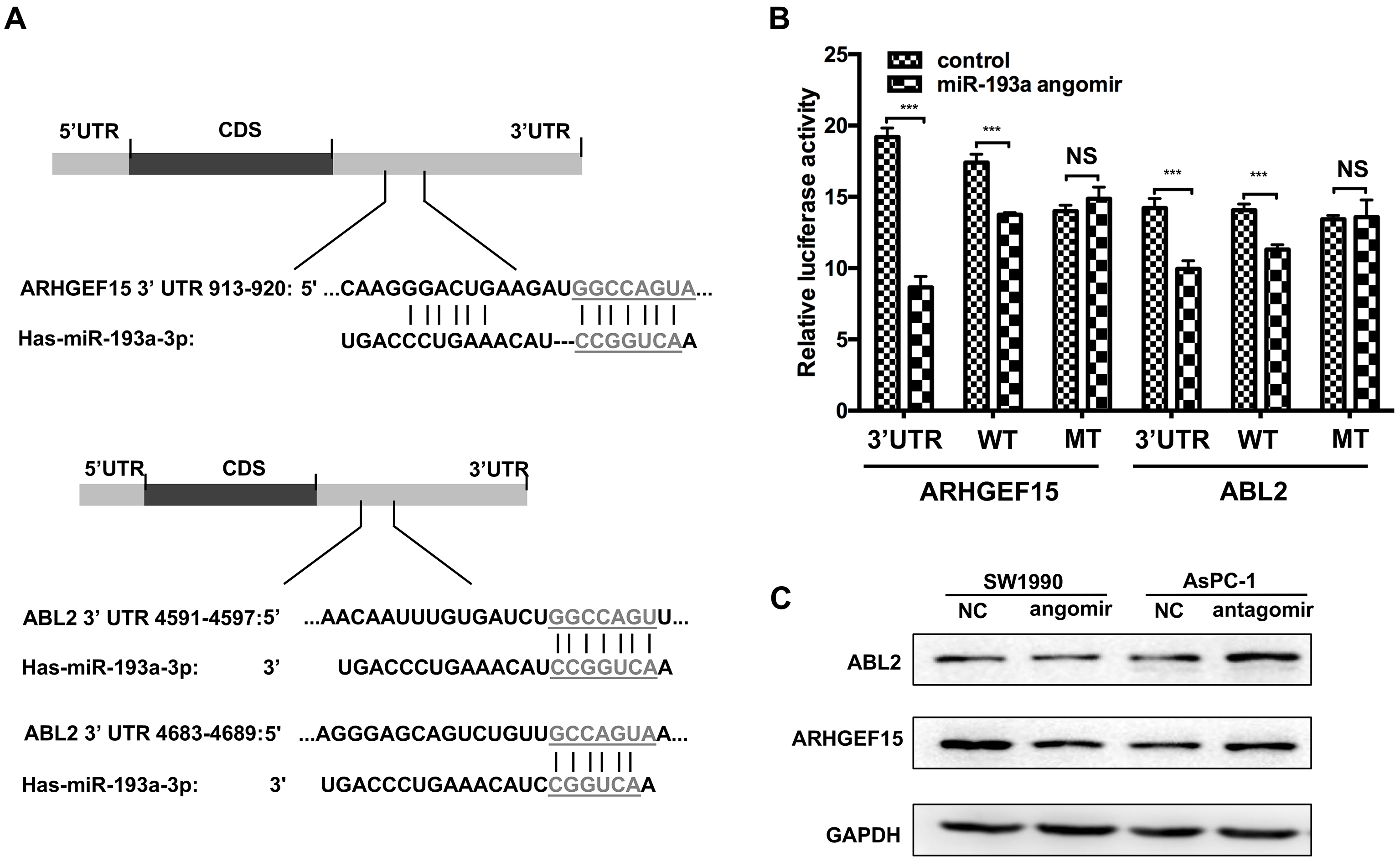

Supplement: Supplementary file 11 — ARHGEF15 and ABL2 were the target genes of miR-193a. (A) Schematic diagrams of the predicted miR-193a-binding sites in 3’-UTR of ARHGEF15 and ABL2. (B) Relative luciferase activity on 3’-UTR, WT and MT counterparts were measured. ***p < 0.001. n = 3. (C) Western blot for detecting ARHGEF15 and ABL2 proteins in SW1990 cells transfected with miR-193a angomir and AsPC-1 cells transfected with miR-193a antagomir. (TIFF 762 kb) [file 13046_2018_697_MOESM11_ESM.tif]

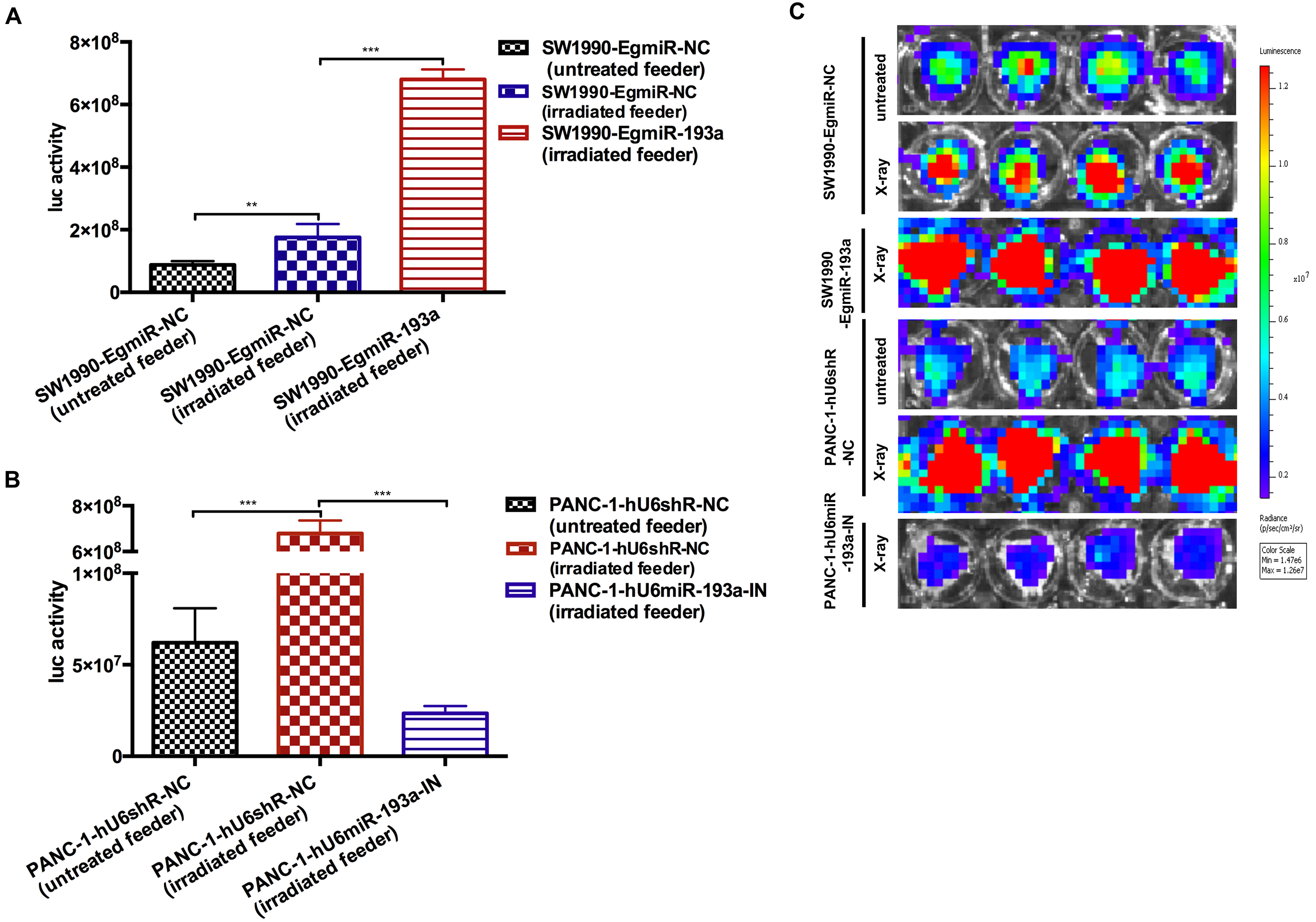

Supplement: Supplementary file 12 — Repopulation of SW1990-EgmiR-NC vs SW1990-EgmiR-193a (A), and PANC-1-hU6shR-NC vs -PANC-1-hU6miR-193a-IN reporter cells (B) co-cultured with corresponding SW1990 and PANC-1 feeder cells. All feeder cells were irradiated with lethal dose (10Gy) except the top line and fourth line. n = 4. The luciferase activity was shown as photons/s. **p < 0.01, ***p < 0.001. (C) Presentative bioluminescence images. (TIFF 1898 kb) [file 13046_2018_697_MOESM12_ESM.tif]
